# The Caprera Canyon (north–eastern Sardinia): A hotspot of cetacean diversity in the western Mediterranean Sea

**DOI:** 10.1371/journal.pone.0326426

**Published:** 2025-07-09

**Authors:** Luca Bittau, Renata Manconi, Mariliana Leotta, Rossana Tenerelli, Mattia Cristina Leone, Elena Fontanesi, Federica Fonda, Ginevra Boldrocchi, Sandro Carniel, Rocco Tiberti

**Affiliations:** 1 SEA ME Sardinia association (Scientific Education and Activities in the Marine Environment), La Maddalena, Italy; 2 Department of Veterinary Medicine, University of Sassari, Sassari, Italy; 3 Delfini del Ponente APS, Imperia, Italy; 4 Department of Life Science, University of Trieste, Trieste, Italy; 5 Department of Human Sciences, Innovation and Territory, University of Insubria, Como, Italy; 6 CNR-ISP, Mestre, Venezia, Italy; 7 One Ocean Foundation, Milano, Italy; 8 Department of Biology, Ecology, and Earth Sciences (DiBEST), University of Calabria, Rende, Cosenza, Italy; University of Minnesota, UNITED STATES OF AMERICA

## Abstract

The Caprera Canyon, a submarine canyon system off Sardinia in the western Mediterranean, was thought to be an important cetacean habitat, though studies on the area remained limited. To address this knowledge gap, 216 boat–based surveys were conducted between 2011 and 2019, covering 8443 km, using both research and whale–watching vessels. The distribution, diversity, relative abundance (Encounter Rate – ER), and habitat use of cetaceans were described, along with relevant behavioural and ecological observations. A total of 8 species were reported across 810 sightings, encompassing 7 out of the 8 cetaceans regularly found in the western Mediterranean Sea. These species, in order of relative abundance, include the striped dolphin (*Stenella coeruleoalba*), fin whale (*Balaenoptera physalus*), Cuvier’s beaked whale (*Ziphius cavirostris*), sperm whale (*Physeter macrocephalus*), Risso’s dolphin (*Grampus griseus*), common dolphin (*Delphinus delphis*), bottlenose dolphin (*Tursiops truncatus*), and a single observation of Sowerby’s beaked whale *Mesoplodon bidens*. This results in an overall ER of 10.6 sightings/100 km. ERs indicate that some species are particularly abundant in the study area, with Cuvier’s beaked whale registering one of the highest ER values ever documented in the Mediterranean Sea. Calves and behavioural observations suggest that the study area serves as both a breeding and feeding ground for most species. The high cetacean diversity and relative abundance, along with the regular occurrence of endangered and vulnerable species, identify the Caprera Canyon as an important hotspot for cetacean diversity. Habitat suitability models using the maximum entropy (MaxEnt) approach highlight that other smaller canyons surrounding the Caprera Canyon could provide suitable habitats for deep diving species. The results presented here serve as reference data for future studies on the ecology and long–term dynamics of cetaceans in the central–western Tyrrhenian Sea. We highlight that the Caprera Canyon and surrounding areas could benefit from being designated as an Important Marine Mammal Area (IMMA), potentially supporting conservation efforts, guiding marine spatial planning, and informing policy development to mitigate threats in the region.

## Introduction

Given their key ecological roles and frequently unfavourable conservation status [1], monitoring and protecting cetacean populations is necessary for maintaining marine ecosystems and biodiversity. However, this endeavour presents significant challenges, particularly due to the high costs associated with dedicated research, which often hinder monitoring efforts [2]. Additionally, the high mobility of cetaceans, combined with limited knowledge of their life history, habitat dynamics, and threats, can reduce the effectiveness of conventional conservation measures, such as establishing Marine Protected Areas (MPAs), which are typically more effective for habitats and sedentary species [3,4].

The Mediterranean Sea covers less than 1% of the Earth’s ocean surface but harbours up to 18% of marine species and is recognized as a biodiversity hotspot [5]. Among marine fauna, cetaceans play vital roles in marine ecosystems as apex consumers, key species, or ecosystem engineers, contributing to processes including iron fertilization and carbon sequestration in the deep sea [6,7]. However, intense human activity along Mediterranean coasts leads to severe and cumulative impacts on marine biodiversity [5,8] and cetaceans, including, for example fisheries bycatch and overfishing, ship strikes, naval sonar use, chemical pollution, and plastic ingestion. Collecting data on cetacean abundance and distribution, even at a local scale, can help identify important suitable areas and highlight potential present and future impacts on both species and ecosystems [3]. However, knowledge gaps persist, particularly in some large areas of the eastern and southern parts of the basin [9], as well as in smaller yet potentially important areas [9,10]. Along with large scale research, small–scale studies, ranging from regional to local frameworks, are therefore necessary to identify critical habitats and relevant areas of conservation for cetaceans [11,12].

To date, the distribution and habitat use have been studied in several areas of the Mediterranean Sea using different research platforms (aerial, ship, and small boat surveys) [13–19]. Many studies have focused on the western basin, where 8 out of the 11 cetacean species regularly inhabiting the Mediterranean Sea have been found to occur year–round [20]: fin whale *Balaenoptera physalus* Linnaeus, 1758, sperm whale *Physeter macrocephalus* Linnaeus, 1758, striped dolphin *Stenella coeruleoalba* (Meyen, 1833), Cuvier’s beaked whale *Ziphius cavirostris* Cuvier, 1823, common bottlenose dolphin *Tursiops truncatus* (Montagu, 1821), common dolphin *Delphinus delphis* Linnaeus, 1758, long–finned pilot whale *Globicephala melas* (Traill, 1809), and Risso’s dolphin *Grampus griseus* (G. Cuvier, 1812). Some of these Mediterranean populations are genetically or ecologically distinct from their north Atlantic conspecifics [20–24]. In this context, studies focusing on submarine canyons, such as the Cuma Canyon in the southern Tyrrhenian Sea [25–27] and the canyon system off the central Catalan coast in the north–western Mediterranean Sea [10], have shown that even relatively small areas can be crucial, as they may serve as important habitats. Seamounts and submarine canyons are known to significantly impact oceanographic and biological processes [28]. Upwelling phenomena associated with submarine canyons enhances local primary productivity, creating bottom–up effects that extend along the food chain up to cetaceans [29–32], influencing their distribution [33] as they can host feeding grounds [34]. Among the system of 29 submarine canyons that incise the Sardinia Island slope, the Caprera Canyon has been identified as an important feeding ground for several marine species (e.g., fish, seabirds, marine turtles) due to the high primary productivity of these pelagic waters [30]. Preliminary regional–scale data [11,35,36] suggested that the geomorphological and oceanographic features of the area could support potentially large aggregations of various cetacean species [37]. However, quantitative data on cetacean distribution and ecology were virtually lacking for the Caprera Canyon and adjacent waters.

The present study aims to assess the conservation importance of the area, addressing the existing gaps in information on cetaceans and providing baseline knowledge of their ecology in the north–eastern Sardinian pelagic waters. Specifically, our objective is to provide a comprehensive description of cetacean distribution, diversity, relative abundance, and habitat use in the Caprera Canyon and surrounding areas. This study represents an essential initial step in understanding and addressing the conservation needs of the cetacean species in the area. Results will also be discussed from a conservation perspective. Notably, in 2016, the Caprera Canyon was officially selected as an Area of Interest (AoI) [38], representing the initial step in its recognition as a potential Important Marine Mammal Area (IMMA) [39]. This designation, introduced in 2016 by the International Union for Conservation of Nature (IUCN) Task Force on Marine Mammal Protected Areas (MMPATF) [39], identifies discrete portions of habitat critical for one or more marine mammal species [40]. However, as a non–legally binding framework, an IMMA alone may not fully protect marine mammals in the Caprera Canyon area or provide immediate conservation benefits. IMMAs are not MPAs and do not include specific conservation measures. Nevertheless, they serve as valuable tools for integrating marine mammal conservation into existing policies and institutional frameworks. Moreover, the broader IMMA network can support the development of future MPA networks [40].

## Materials and methods

### Study area

**Fig 1:**
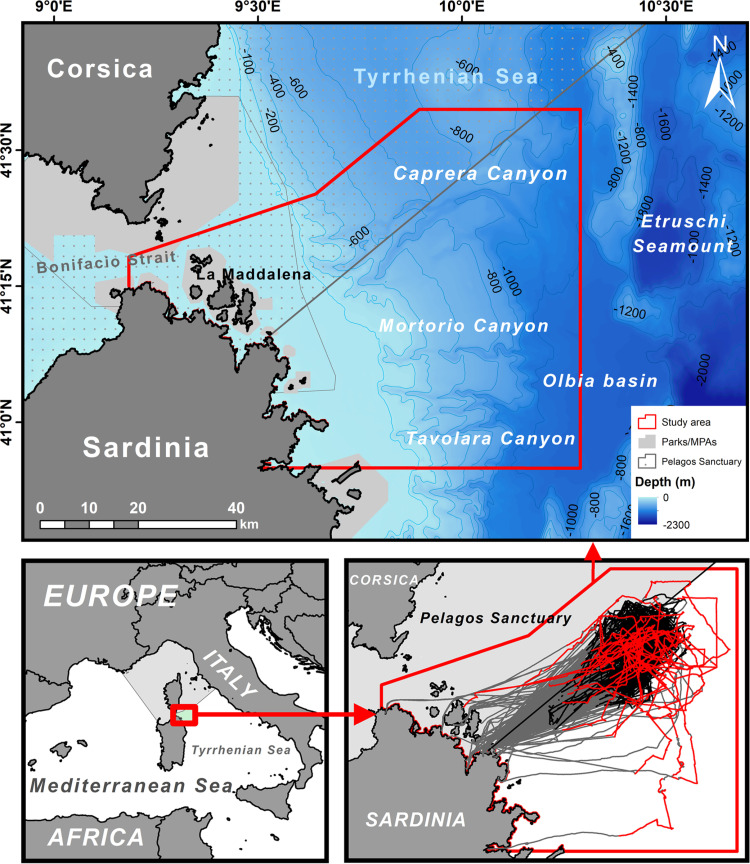
Study area. (*Top*) Map of the Caprera Canyon and surrounding waters, with bathymetry represented by varying shades of blue and isobaths indicated. The nearby national/international parks are shown in light grey, and the Pelagos Sanctuary with a dotted area. (*Bottom left*) Location of the study area in the Mediterranean Sea. (*Bottom right*) Search effort tracklines offshore Sardinia 2011–2019, with overall tracklines (light grey) and on–effort tracklines (black = platform of opportunity; red = dedicated research vessels).

The study area is located in and around the Caprera Canyon, east of the Bonifacio Strait (central–western Tyrrhenian Sea), extending to 10°17’E and spanning between 40°54’N and 41°34’N (Fig 1). It is characterized by a complex seabed physiography, including continental shelf and slope, canyons, and seamounts, with depths reaching up to 1500 m. The continental shelf is quite wide, extending 20 km off the north–eastern coast of Sardinia, extending into a 17–26 km wide continental slope at around 120 m depth. The slope, narrow and steeper in the southern sector and wider with a gentler slope in the north, descends to approximately 1200 m. Beyond this, the Olbia Basin plain extends to 1650 m depth, bounded to seaward by the Etruschi seamounts (Fig 1). The north–eastern Sardinian slope is also deeply incised by several turbidite canyon systems, including the Caprera, Mortorio, and Tavolara canyons, with the Caprera Canyon being the major system (Fig 1). The Caprera Canyon consists of 2 meandering tributary canyons, each approximately 16 km long, incised in the upper continental slope. These canyons merge into a single 2.5 km–wide canyon at around 1000 m depth in the lower slope and extend for a further 9 km toward the continental rise.

At a regional scale, sea circulation is dominated by the Tyrrhenian Gyre, a large cyclonic system [41]. The combination between the wind driven current outflowing from the Bonifacio Strait and the predominant current flowing southward, along the Corsican Tyrrhenian coast, forms two main eddies: a permanent cyclonic eddy (“Bonifacio Cyclone”) and an adjacent, southern anticyclonic eddy off the Sardinian eastern coast [42]. The Bonifacio eddy, combined with seabed topography, drives the upwelling of cold, deep water [43,44], enhancing local productivity in the region, including within the Caprera Canyon system [30].

From a conservation perspective, the Caprera Canyon area is located just outside, but in close proximity to, the south–eastern border of the Pelagos Sanctuary (Fig 1). The International Sanctuary for the Protection of Mediterranean Marine Mammals, known as the Pelagos Sanctuary, was established in 1999 through an agreement between Italy, France and the Principality of Monaco. In 2001, it was designated as a Specially Protected Area of Mediterranean Importance (SPAMI) [45,46]. The Caprera Canyon is part of the north–western Mediterranean Pelagic Ecosystems EBSA (Ecologically or Biologically Significant Area), proposed by UNEP [37]. Since 2012, it has also been included in the Ecological Protection Zone (EPZ) under Italian law (D.P.R. 27/10/2011 no. 209). However, this designation primarily extended national jurisdiction beyond territorial waters rather than implementing effective protection measures.

Additionally, the Caprera Canyon area is located near several protected areas, including: La Maddalena Archipelago National Park (Italy), the Bouches de Bonifacio Natural Reserve SPAMI (France), and various Special Areas of Conservation (SACs) under the European Union’s Habitats Directive (92/43/EEC), such as La Maddalena (ITB010008), Capo Testa–Isola Rossa (ITB013052), Bouches de Bonifacio (FR9402015), Iles Des Moines, and Iles Cerbicale (FR9410022) (Fig 1). In 2011, the International Maritime Organization (IMO) designated the Bonifacio Strait and its adjacent waters as the first Particularly Sensitive Sea Area (PSSA) in the Mediterranean [47]. This designation highlights maritime safety and environmental protection due to the area’s ecological significance, uniqueness, high maritime traffic volume, and vulnerability to maritime accidents and pollution. However, this network of marine protected areas primarily encompasses continental shelf habitats and does not include the Caprera Canyon system. Despite its inclusion in or proximity to several protected areas, no specific conservation measures address cetacean populations in this pelagic region.

### Boat survey and sighting protocols

Most cetacean surveys (96%) were conducted using platforms of opportunity, such as whale–watching. The remaining surveys were carried out from dedicated research vessels, planned to cover some less–surveyed areas distant from typical whale–watching routes distributed around the canyon area (Fig 1). Despite some shortcomings associated with platforms of opportunity [48], they are commonly and effectively used in cetacean monitoring [49,50], overcoming the high navigation costs associated with cetacean research in offshore areas. Five boats were used for cetacean surveys during the study period: three 11 m long power catamarans and two 9–10 m long monohull motorboats. All boats were equipped with an observation platform (fly–bridge or observation tower) positioned at a height ranging from 4.3 to 5 meters above sea level (depending on the vessel used), ensuring a similar range of observation across all vessels.

The data collection protocol remained consistent throughout the 9–year study period, regardless of whether surveys were opportunistic or dedicated. Field protocols, slightly adapted from our colleagues [51–53], are described in detail in the Supporting Information (S1 File). In summary, visual boat surveys for cetaceans were conducted in favourable sea and weather conditions along line transects, with at least three experienced observers using 7x50 compass binoculars. Environmental, positional and sighting data were recorded on standardized sheets or audio devices, with GPS positions and navigation details logged every 5 minutes. GPS data were downloaded using Garmin BaseCamp (v4.7.4) and stored in a ESRI’s ArcGIS© Geodatabase (ESRI, Redlands, California). Surveys had two modes: ‘on–effort’, conducted under favourable conditions (±30 minutes from dusk/dawn, Beaufort ≤3, Douglas ≤2, visibility ≥5 km, swell ≤0.5 m, no rain, vessel speed 6–10 knots and at least 2 observers working at their stations), and ‘off–effort’, when any of these conditions were not met or during sightings. The overall on–effort portion of a survey was considered the sum of all boat track–line sections meeting these criteria. During sightings, the survey mode switched to off–effort. Observers recorded cetacean positions, species ID, group size (average of observers’ estimates), age class (when identifiable), presence of calves/newborn and behavioural data. The initial sighting position was estimated based on sighting distance and bearing angle from the vessel. These positions were used to unambiguously define the geographic location of each sighting. A sighting of delphinids or beaked whales was defined as a positive detection of individuals engaged in the same activity and in apparent association, typically moving in the same direction, within a 0.5 km radius [54–56]. For large cetaceans, a group was considered as one or more individuals remaining in coordinated behaviour throughout the sighting, within a 1.0 km radius for fin whales (modified from our colleagues [56]). For sperm whales, we adopted the definitions of an “aggregation group”, according to our colleagues [57].

### Cetacean community composition, diversity, encounter rates, distribution, and habitat suitability

A list of the sighted cetacean species was provided, along with the relative contribution of each species to the cetacean community, expressed as percent sighting frequency (SFi = n_*i*_ / N × 100), where n_*i*_ is the number of on–effort sightings of each species i, divided by the total number of on–effort sightings of all species in the study area.

To describe the diversity of the cetacean community in the Caprera Canyon area, some common diversity indices were used including species richness (S), the Shannon–Wiener index (H’), and the Evenness index (E) [58–60]. All sighting records, including off–effort sightings, were used to calculate these indices, to account for species that were rarely or never observed during on–effort surveys. To understand the spatial distribution of the different species and estimate their relative abundance, we calculated the encounter rate (ER = n_*i*_ / L × 100 km), where n_*i*_ represents the number of on–effort sightings of distinct groups of cetaceans of each species *i*, and L is the length in kilometres of survey travelled on–effort [61]. The ERs were computed separately for each survey, considering each survey as an independent sampling unit.

Cetacean distribution in the study area was described through a map for each species. To investigate the influence of key habitat features on the distribution of the most common cetacean species and to assess the suitability of adjacent unsampled areas, habitat suitability was modelled using the MaxEnt algorithm [62] based on the on–effort sighting data. The main output of MaxEnt is a map representing the suitability of habitats through a continuous index varying between 0 (very unsuitable habitats) and 1 (very suitable habitats). This is a widely used statistical modelling approach which applies the principle of maximum entropy to predict the potential distribution of species from presence–only data in relation to spatial predictor variables [63]. Some important topographic variables were included as predictors: depth, slope, and aspect derived from a Digital Elevation Model (DEM with a resolution of 200 m). These were resampled into a grid with 2x2 km cell size and rasterized, using QuantumGIS 3.18.3. Aspect was converted into 8 raster grids representing the 8 facing positions of the slope (N, NE, E, SE, S, SW, W, NW), which were used as predictors. Moreover, the presence of the submerged canyons in the habitat suitability analysis was taken into account, considering their importance for the distribution and abundance of cetaceans [31]. To this end, the Topographic Position Index (TPI; [64]) was computed using SAGA GIS with a radius of 2 km, and it was used to identify the canyon–like features as described by [64]. All minor features and artefacts smaller than 3 km were eliminated [65]. The distance of these canyon–like features was calculated and used as predictor variable for the models. Multi–collinearity among variables was tested through the Variance Inflation Factor (VIF) with a threshold of 3, VIF > 3 indicating highly correlated predictors [66]. Given that the MaxEnt algorithm has shown particular efficiency with small samples [67], it was decided to fit models for species with more than 10 presence data/occurrences/sightings on–effort. These species include the striped dolphin (*S. coeruleoalba*), fin whale (*B. physalus*), Cuvier’s beaked whale (*Z. cavirostris*) and sperm whale (*P. macrocephalus*). Because the non–random distribution of survey effort in the study area may lead to biased predictions [68], a sampling effort map was created by generating a Gaussian Kernel Density map of the vessel survey/track–line/transect [69]. Then, randomly sampled background points (n = 5000) were superimposed to this bias map (S1 Fig). To ensure more ecologically realistic response curves, MaxEnt was run using only linear and quadratic features [70] and default values for all the other parameters (max number of iterations = 5000; convergence threshold = 10–5; multiplier regularization = 1) [63]. For each MaxEnt model, 10 replicates were run with tenfold cross validation, and their average was taken as the final model [71]. The relative contribution of each variable was assessed using a jackknife [72] and the best–fitting model was obtained by removing all variables with no contribution, calculated as the arithmetic mean between the percent contribution and permutation importance, < 5% [73]. Model accuracy was analysed by the area under the curve of the receiver operating characteristic (ROC) [74]. All statistical analyses were carried out on R v. 4.0.1 (R Core Team, 2021) using the packages dismo [75], usdm [76] and ENMeval [77].

## Results

### Survey efforts

A total of 216 one–day boat surveys (total 29175 km; 20732 km covered off–effort and 8443 km on–effort) were performed between 2011 and 2019. Of these, 8 were conducted with dedicated research vessels, and the remaining 208 were carried out with whale–watching vessels.

**Fig 2:**
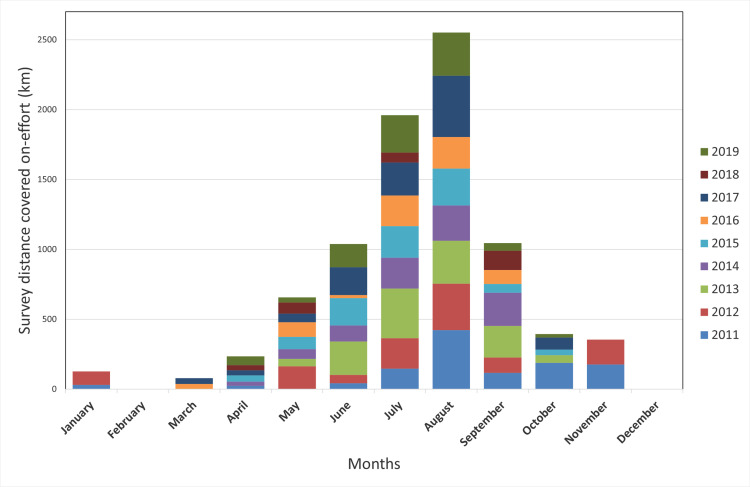
Monthly survey effort (on–effort km) from 2011 to 2019. Survey effort, represented as stacked bars, with different colours indicating contributions from each year. Survey coverage was highest during the summer months (July and August), with reduced effort in winter and early spring. The variability in effort reflects seasonal and logistical constraints associated with data collection.

On–effort routes covered a wide area off north–eastern Sardinia, characterized by complex topography, from the continental shelf to the deep pelagic waters of the middle bathyal zone. Most of the effort has focused on upper and lower slope habitats, while continental shelf and rise habitats in the study area remained poorly or not surveyed (Fig 1). Survey effort remained relatively consistent across years, with most surveys conducted from May to October (Fig 2), encompassing late spring, summer, and autumn, while only a few surveys were performed during winter and spring.

### Sighting summary

A total of 1110 cetacean sightings (810 on–effort) were recorded, corresponding to 11867 sighted individuals (8747 sighted on–effort; see Table 1), excluding sightings with incomplete data and uncertain species identification. A detailed species–by–species sighting summary, including distribution, behavioural and observational data, as well as photographic documentation, is available in the Supporting Information (S2 File). Below, we provide a concise overview of the sighting data.

**Table 1:** Summary of sighting, survey and diversity data, including encounter rates (ER), group size, depth and slope relative to cetaceans in the Caprera Canyon waters between 2011 and 2019.

	2011		2012		2013		2014		2015		2016		2017		2018		2019		Total	
**SURVEY DATA**																				
Survey period	16/01–27/11		18/01–24/11		16/03–09/10		14/04–28/09		15/04–25/10		19/03–23/09		12/03–17/10		22/04–16/09		01/04–12/10			
No. of surveys	28		25		33		27		26		22		27		7		21		**216**	
Tot. Km traveled	3774.7		3634.1		4284.7		3506.0		3447.9		2885.7		3688.3		1010.7		2942.5		**29175**	
Tot. Km travelled on-effort	1147.0		1155.7		1234.7		927.3		919.7		710.5		1099.2		325.3		923.4		**8443**	
**SPECIES**																				
*S. coeruleoalba*																				
Sightings	83	(99)	83	(105)	55	(73)	44	(67)	39	(64)	46	(65)	47	(65)	18	(24)	32	(42)	**447**	**(604)**
No. Encountered individuals	1059	(1285)	1459	(1799)	1526	(1813)	768	(1197)	710	(1023)	889	(1164)	730	(946)	242	(404)	602	(801)	**7985**	**(10432)**
Group size, mean (±SD)	12.98	(10.87)	17.13	(14.10)	24.84	(19.14)	17.87	(12.29)	15.98	(12.00)	17.91	(14.60)	14.55	(11.83)	16.83	(14.72)	19.07	(15.84)	**17.27**	**(14.24)**
Encounter rate, mean (±SD)	**7.71**	**(5.47)**	**7.01**	**(4.28)**	**4.67**	**(3.71)**	**4.80**	**(6.02)**	**4.50**	**(3.82)**	**6.91**	**(6.35)**	**4.40**	**(3.96)**	**5.55**	**(2.45)**	**3.56**	**(3.00)**	**5.45**	**(4.76)**
Depth (m) overall sgts., mean (±SD)	786	(154.7)	815	(160.2)	776	(136.5)	800	(138.3)	788	(161.4)	806	(140.6)	797	(136.0)	710	(154.0)	807	(139.7)	**793**	**(148.6)**
Depth (m) on-effort sgts., mean (±SD)	796	(147.3)	835	(155.7)	806	(119.0)	835	(89.7)	834	(98.1)	859	(101.2)	833	(96.1)	745	(126.7)	856	(112.1)	**824**	**(126.1)**
Slope (%), mean (±SD)	5.5	(4.9)	5.7	(5.1)	3.6	(4.0)	4.8	(4.9)	5.6	(5.0)	5.8	(5.3)	6.6	(5.5)	5.9	(5.6)	3.9	(4.1)	**5.3**	**(5.0)**
*B. physalus*																				
Sightings	40	(44)	22	(25)	37	(46)	39	(47)	17	(20)	15	(16)	19	(23)	14	(20)	3	(3)	**206**	**(244)**
No. Encountered individuals	63	(68)	37	(42)	60	(70)	67	(80)	26	(32)	27	(28)	29	(35)	22	(31)	3	(3)	**334**	**(389)**
Group size, mean (±SD)	1.55	(0.70)	1.68	(0.75)	1.52	(0.94)	1.70	(0.81)	1.60	(0.68)	1.75	(0.86)	1.52	(0.79)	1.55	(0.83)	1	(0)	**1.59**	**(0.79)**
Encounter rate, mean (±SD)	**4.11**	**(3.78)**	**2.53**	**(3.21)**	**3.04**	**(3.12)**	**5.14**	**(5.84)**	**2.86**	**(4.99)**	**2.47**	**(2.77)**	**2.15**	**(4.75)**	**5.06**	**(5.93)**	**0.41**	**(1.07)**	**3.00**	**(4.20)**
Depth (m) overall sgts., mean (±SD)	767	(278.5)	756	(210.4)	802	(114.6)	845	(150.2)	877	(97.8)	947	(107.0)	810	(208.9)	754	(228.0)	832	(5.6)	**812**	**(192.4)**
Depth (m) on-effort sgts., mean (±SD)	780	(270.1)	818	(89.8)	824	(89.3)	876	(96.4)	881	(102.7)	954	(106.5)	875	(126.0)	850	(120.7)	832	(5.6)	**845**	**(154.8)**
Slope (%), mean (±SD)	5.9	(5.0)	3.4	(2.6)	5.2	(5.8)	4.9	(4.1)	6.4	(5.9)	7.5	(7.4)	5.6	(5.4)	6.2	(4.8)	2.0	(1.7)	**5.4**	**(5.1)**
*Z. cavirostris*																				
Sightings	9	(9)	21	(22)	29	(33)	14	(18)	12	(13)	19	(22)	15	(19)	1	(1)	10	(12)	**130**	**(149)**
No. Encountered individuals	21	(21)	37	(38)	72	(81)	31	(42)	27	(29)	48	(54)	31	(39)	4	(4)	21	(25)	**292**	**(333)**
Group size, mean (±SD)	2.33	(0.87)	1.73	(0.83)	2.45	(1.00)	2.33	(1.37)	2.23	(1.37)	2.45	(2.09)	2.05	(0.85)	4	(0)	2.08	(0.79)	**2.23**	**(1.20)**
Encounter rate, mean (±SD)	**1.09**	**(1.87)**	**2.07**	**(2.59)**	**2.44**	**(3.14)**	**1.57**	**(1.98)**	**1.39**	**(2.24)**	**3.75**	**(6.99)**	**1.36**	**(1.60)**	**0.34**	**(0.90)**	**1.18**	**(1.57)**	**1.80**	**(3.12)**
Depth (m) overall sgts., mean (±SD)	842	(89.5)	900	(101.8)	854	(67.4)	866	(93.7)	846	(76.9)	868	(111.8)	895	(83.3)	-		830	(69.7)	**866**	**(88.5)**
Depth (m) on-effort sgts., mean (±SD)	842	(89.5)	901	(104.0)	854	(69.6)	879	(102.9)	851	(78.5)	882	(112.6)	881	(82.3)	-		829	(71.8)	**869**	**(90.4)**
Slope (%), mean (±SD)	6.6	(3.8)	6.8	(5.1)	3.5	(3.4)	6.9	(5.2)	3.5	(3.9)	6.3	(7.4)	7.9	(7.5)	-		3.9	(2.7)	**5.5**	**(5.3)**
*P. macrocephalus*																				
Sightings	-		1	(1)	8	(9)	1	(1)	1	(1)	-	(1)	-		1	(1)	1	(1)	**13**	**(15)**
No. Encountered individuals	-		3	(3)	27	(28)	1	(1)	4	(4)	-	(2)	-		1	(1)	1	(1)	**37**	**(40)**
Group size, mean (±SD)	-		-		3.11	(4.20)	1	(0)	4	(0)	2	(0)	-		1	(0)	1	(0)	**2.67**	**(3.31)**
Encounter rate, mean (±SD)	**-**		**0.10**	**(0.52)**	**0.67**	**(1.22)**	**0.10**	**(0.53)**	**0.16**	**(0.81)**	**-**		**-**		**0.34**	**(0.90)**	**0.13**	**(0.61)**	**0.17**	**(0.69)**
Depth (m) overall sgts., mean (±SD)	-		-		776	(108.9)	-		-		-		-		-		-		**764**	**(103.4)**
Depth (m) on-effort sgts., mean (±SD)	-		-		798	(92.8)	-		-		-		-		-		-		**784**	**(95.2)**
Slope (%), mean (±SD)	-		-		4	(4.1)	-		-		-		-		-		-		**5.6**	**(5.1)**
*T. truncatus*																				
Sightings	1	(7)	2	(12)	-	(19)	-	(11)	-	(8)	-	(5)	-	(11)	1	(3)	-	(3)	**4**	**(79)**
No. Encountered individuals	2	(21)	18	(67)	-	(100)	-	(45)	-	(26)	-	(12)	-	(35)	2	(15)	-	(18)	**22**	**(339)**
Group size, mean (±SD)	3.00	(1.83)	5.58	(3.90)	5.26	(6.49)	4.09	(3.21)	3.25	(2.43)	2.40	(1.52)	3.18	(2.04)	5.00	(4.36)	6.00	(2.00)	**4.29**	**(4.06)**
Encounter rate, mean (±SD)	**0.10**	**(0.55)**	**0.11**	**(0.39)**	**-**		**-**		**-**		**-**		**-**		**0.18**	**(0.47)**	**-**		**0.03**	**(0.25)**
Depth (m) overall sgts., mean (±SD)	102	(60.1)	154	(136.3)	111	(98.0)	73	(30.5)	95	(50.0)	91	(4.4)	96	(47.9)	90	(9.3)	97	(20.2)	**105**	**(79.6)**
Depth (m) on-effort sgts., mean (±SD)	-		368	(202.94)	-		-		-		-		-		-		-		**262**	**(178.2)**
Slope (%), mean (±SD)	-		10	(5.7)	-		-		-		-		-		-		-		**6.3**	**(5.8)**
*G. griseus*																				
Sightings	2	(2)	2	(2)	-		-		2	(2)	2	(3)	1	(1)	-		-		**9**	**(10)**
No. Encountered individuals	16	(16)	25	(25)	-		-		16	(16)	9	(12)	10	(10)	-		-		**76**	**(79)**
Group size, mean (±SD)	8.00	(7.07)	12.50	(0.71)	-		-		8.00	(8.49)	4.00	(1.00)	10	(0)	-		-		**7.90**	**(4.91)**
Encounter rate, mean (±SD)	**0.22**	**(0.81)**	**0.19**	**(0.67)**	**-**		**-**		**0.17**	**(0.59)**	**0.31**	**(1.02)**	**0.11**	**(0.55)**	**-**		**-**		**0.12**	**(0.57)**
Depth (m) overall sgts., mean (±SD)	725	(40.3)	819	(4.2)	-		-		771	(24.0)	835	(200.9)	-		-		-		**796**	**(106.0)**
Depth (m) on-effort sgts., mean (±SD)	725	(40.3)	819	(4.2)	-		-		771	(24.0)	939	(126.6)	-		-		-		**815**	**(93.0)**
Slope (%), mean (±SD)	5	(1.4)	2	(0.7)	-		-		6	(4.9)	7	(7.8)	-		-		-		**4.3**	**(3.9)**
D. delphis*																				
Sightings	-		-		-	(2)	-		-	(1)	-	1	-	(3)	-	(1)	-		**-**	**(8)**
No. Encountered individuals	-		-		-	(80)	-		-	(35)	-	25	-	(54)	-	(60)	-		**-**	**(254)**
Group size, mean (±SD)	-		-		40.00	(28.28)	-		35	(0)	25	(0)	18.00	(10.39)	60	(0)	-		**31.75**	**(19.17)**
Encounter rate, mean (±SD)	**-**		**-**		**-**		**-**		**-**		**-**		**-**		**-**		**-**		**-**	
Depth (m) overall sgts., mean (±SD)	-		-		590	(37.5)	-		-		-		618	(114.5)	-		-		**551**	**(145.0)**
Depth (m) on-effort sgts., mean (±SD)	-		-		-		-		-		-		-		-		-		**-**	
Slope (%), mean (±SD)	-		-		1	(1.4)	-		-		-		10.3	(9.7)	-		-		**8.1**	**(7.8)**
**Total Encounter rate, mean (±SD)**	**13.24**	**(7.50)**	**12.16**	**(5.73)**	**10.81**	**(5.59)**	**11.61**	**(9.72)**	**9.08**	**(7.20)**	**13.44**	**(9.21)**	**8.01**	**(6.77)**	**11.47**	**(6.76)**	**5.27**	**(3.50)**	**10.57**	**(7.42)**
**DIVERSITY (Overall sightings)**																				
Species Richness (S)	5		7		6		5		7		7		6		6		5		**8**	
Shannon index (H)	0.41		0.43		0.70		0.51		0.57		0.49		0.66		0.76		0.27		**0.56**	
Simpson index (1-D)	0.17		0.17		0.30		0.23		0.23		0.19		0.28		0.37		0.11		**0.22**	
Evenness (E)	0.25		0.22		0.39		0.31		0.29		0.25		0.37		0.43		0.17		**0.27**	

Data referred to overall sightings and Nº of individuals is shown in brackets.

*Data shown on *Delphinus delphis* refers only to off–effort sightings.

**Fig 3:**
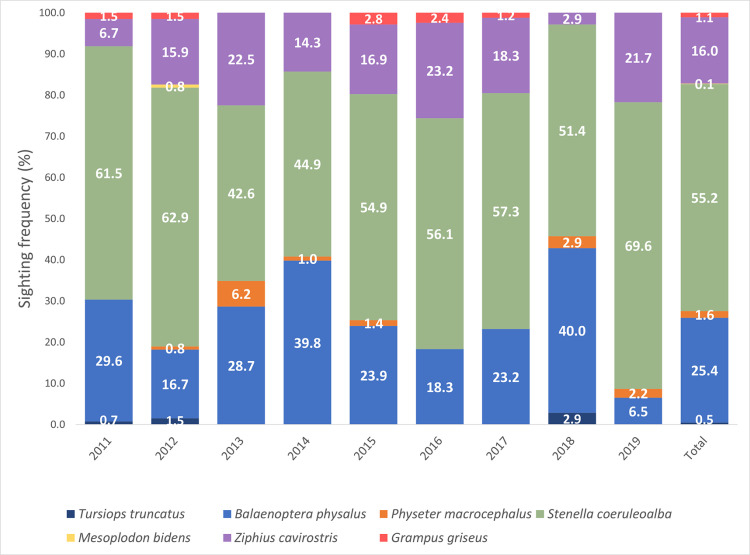
Annual sighting frequency (%) of different cetacean species in the Caprera Canyon and adjacent waters from 2011 to 2019 (n **= 810, on–effort sightings).** The overall sighting frequency for the entire study period (Total) is indicated. Species are represented by different colours.

**Fig 4:**
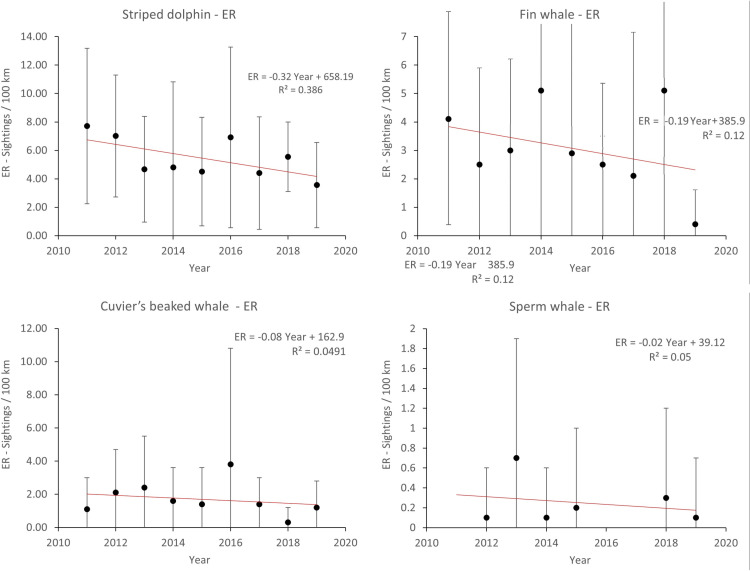
Interannual variability of ER (Mean **± SD) of the most common cetacean species.** The regression line is fitted based on the means of the annual ERs.

Overall, 8 species of cetaceans were identified. Listed in order of encounter rate (ER mean ± SD values reported), sighting frequency (SF; Fig 3), and number of on–effort sightings: striped dolphin (ER = 5.45 ± 4.76; SF = 55.2%; n = 447), fin whale (ER = 3.00 ± 4.2; SF = 25.4%; n = 206), Cuvier’s beaked whale (ER = 1.80 ± 3.12; SF = 16.0%; n = 130), sperm whale (ER = 0.17 ± 0.69; SF = 1.6%; n = 13), Risso’s dolphin (ER = 0.12 ± 0.57; SF = 1.1%; n = 9), common bottlenose dolphin (ER = 0.03 ± 0.25; SF = 0.5%; n = 4), common dolphin (sighted only off–effort, 8 times), and Sowerby’s beaked whale *Mesoplodon bidens* (Sowerby, 1804) (sighted only once, off–effort, in a mixed species group with 3 Cuvier’s beaked whales, [78], S2 File). Except for the latter species, the remaining species were regularly or repeatedly observed over the study period (Table 1, Fig 4, S2 File). Species richness ranged from 5 to 7 species sighted per year. Overall indices indicate moderate diversity (H’ = 0.56) and evenness (E = 0.27). All species persisted throughout the study period (Fig 4).

**Fig 5:**
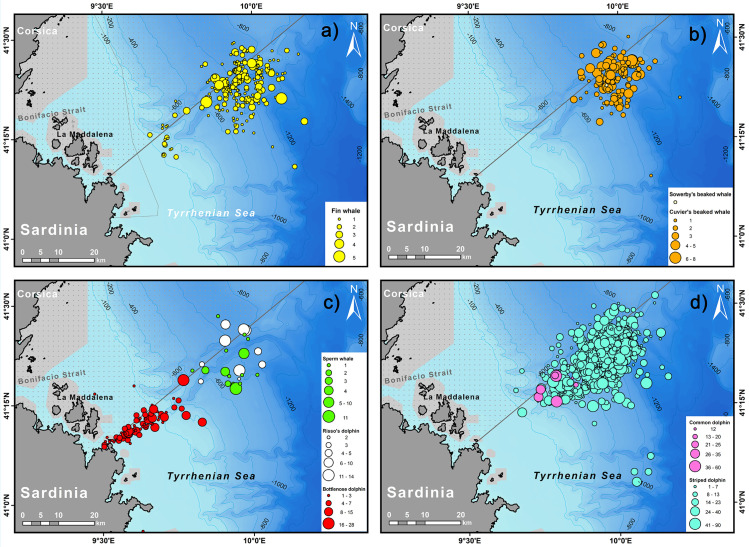
Maps showing location of the cetacean sightings off north–eastern Sardinia, throughout the study period (2011–2019) scaled by group size. The nearby national/international parks are shown in light grey, and the Pelagos Sanctuary as a dotted area. Data shown in the maps are based on 1110 sightings, both ‘on–effort’ and ‘off–effort’. Striped dolphin (n = 604); Fin whale (n = 244); Cuvier’s beaked whale (n = 149); Common bottlenose dolphin (n = 79); Sperm whale (n = 15); Risso’s dolphin (n = 10); Common dolphin (n = 8) and Sowerby’s beaked whale (n = 1).

The striped dolphin was by far the most common species in the study area (Fig 4), forming the largest cetacean groups (group size range, GSR = 1–90 individuals; Fig 5). These groups typically included juveniles and accounted for 30.1% of the sightings with calves. An interesting observation was made of a few–hour old newborn on 17 August 2013 (photographic documentation in S2 File). The common dolphin was sighted only when off–effort, but it formed relatively large groups (GSR = 12–60; Fig 5), including juveniles and calves (observed in 3 out of the 8 sightings), with calves also showing foetal folds (photographic documentation in S2 File). Bottlenose dolphins were mainly observed off–effort and formed relatively small groups (GSR = 2–12; Fig 5) including juveniles and calves. Risso’s dolphins were recorded in pairs or up to 14 individuals, with calves present in 40% of the overall sightings, often with foetal folds (photographic documentation in S2 File). Cuvier’s beaked whales were observed alone in 29.5% of the sightings or in groups of 2 (36.9%), 3 (20.1%), and groups composed of more than 4 and up to 9 individuals accounted for 13.4% of the sightings (Fig 5). Calves were recorded in 18% of the sightings, often with foetal folds. The sperm whale also showed a wide overall group size range (GSR = 1–11; Fig 5). Most sperm whale sightings were of solitary individuals (n = 10), but 1 bachelor group (3 individuals), 1 adult–calf pair, and larger groups were also observed. Two sightings of social units (10 and 11 individuals, respectively) were also observed in July 2013, comprising females, calves and young individuals (Fig 5, S2 File). The observation of a surface–nursing episode (photographic documentation in S2 File), as well as the close association of calves (6–7 m long) with adults, and the simultaneous presence of several individuals within a radius of less than 500 m allowed us to confirm the sightings of social units. Fin whales were observed in groups of up to 5 individuals, but primarily as solitary individuals (54.9% of sightings) or in pairs (32%), with 4 sightings documenting female–calf pairs in close association. Mixed species groups were uncommon: striped dolphin with fin whale (n = 4), striped dolphin with common dolphin (n = 3), Cuvier’s beaked whale with Sowerby’s beaked whale (n = 1) and Cuvier’s beaked whale with fin whale (n = 1). In general, female–calf pairs were frequently observed for most species (striped dolphin, Risso’s dolphin, common dolphin, sperm whale, Cuvier’s beaked whale and bottlenose dolphin).

## Distribution

**Fig 6:**
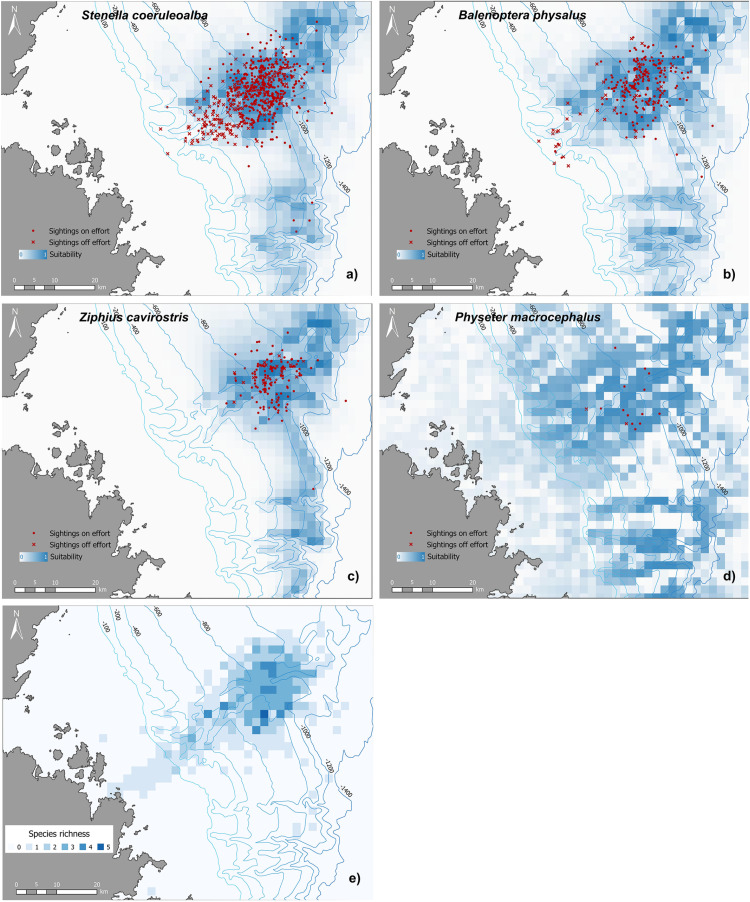
Habitat suitability maps and species richness. Habitat suitability of (a) striped dolphin, (b) fin whale, **(c)** Cuvier’s beaked whale, (d) sperm whale. In the scale bar, index is continuous from very suitable habitats (value 1, dark blue) to very unsuitable habitats (value 0, white) for the species; **(e)** Spatial distribution of species richness, with a region around the Caprera Canyon system (lower slope habitat) of high diversity of cetaceans, where three or more species are present.

The bottom depth relative to the whole dataset at on–effort sighting positions ranged from 87 to 1269 m. Habitat types surveyed on–effort within the study area mainly include the lower slope (95.8% of the on–effort routes), the upper slope (2.2%), and to a lesser extent, areas along the rise (1.3%), within the bathyal zone. Considering the overall sightings (both on– and off–effort records), it is evident that most species in the Caprera Canyon and surrounding areas are associated with pelagic habitats. These habitats support a more diverse community compared to that is observed over the continental shelf, as shown in Fig 5. Among the species observed, only the bottlenose dolphin, primarily recorded off–effort, shows a preference for shallow waters and coastal areas, being virtually absent beyond the continental shelf (mean depth ± SD = 105 m ± 79.6; Fig 5). In contrast, sightings of striped dolphin were recorded beyond the continental shelf, both in the upper (2.7% of sightings) and lower (96.6%) continental slope, at a mean depth of 793 m ± 148.6 (mean ± SD) and over a relatively wide slope range (0–24%) (Fig 5; Table 2). Markedly fewer observations of common dolphin (only in off–effort mode) also come from the continental slope, primarily from the upper slope and at the head of the Caprera Canyon system, at a mean depth ± SD of 551 m ± 145 and a relatively wide slope range (0–21%) (Fig 5; Table 2). Fin whale sightings were primarily recorded in the upper slope (mean depth ± SD = 812 ± 192.4). However, sightings above the continental shelf were frequent, as evidenced by the wide depth range (100–1269 m). Moreover, fin whale showed to occur in a wide slope range (0–24%) (Fig 5; Table 2). The mean depths (± SD) and depth ranges of teuthophagous species, i.e., sperm whale (764 m ± 103.4; range: 657–938 m), Risso’s dolphin (796 m ± 106; range: 696–1028 m), and Cuvier’s beaked whale (866 m ± 88.5; range: 669–1249 m), partially overlap in the lower slope, but the Cuvier’s beaked whale was more frequently sighted over deeper waters (Figs 5 and 6). Cuvier’s beaked whale and sperm whale showed similar mean slopes (Table 1), whereas sperm whale and Risso’s dolphin had comparable slope range (1–16% and 1–12%, respectively). In contrast, Cuvier’s beaked whale sightings exhibited the widest slope range (0–23%).

**Table 2:** Results of the best fitting MaxEnt models. The values in the table represent the arithmetic mean of percent contribution and permutation importance. Higher values indicate greater variable importance.

Variables	*S. coeruleoalba*	*B. physalus*	*Z. cavirostris*	*P. macrocephalus*
Depth	^2^ (47.15)	^2^ (38.70)	^2^ (64.90)	^2^ (15.70)
Distance from canyon–like features	– (22.30)	– (23.60)	– (8.95)	– (12.55)
Slope	– (11.05)	– (8.15)	– (5.20)	
North	^2^ (11.80)			
East		– (22.00)		– (61.25)
South–East		^2^ (7.55)		
South	^2^ (7.65)		+ (21.00)	
South–West				– (10.50)

Effects (“+”: positive; “–”: negative; ^“2”^: quadratic) and contribution (in brackets) of each variable.

### Habitat suitability

Only striped dolphin, fin whale, Cuvier’s beaked whale, and sperm whale had enough on–effort sightings for habitat suitability models and maps to be drawn up (Fig 6). The north–east exposure variable was excluded from all the models to avoid multi–collinearity. AUC values around 0.8 (Striped dolphin AUC = 0.82; fin whale AUC = 0.82; Cuvier’s beaked whale AUC = 0.86; sperm whale AUC = 0.78) indicated that the discriminatory ability of the best models was good [79]. The habitat suitability of all considered species was significantly influenced by bathymetry, with each species showing preferred depth ranges (Table 2, S2 Fig). The maximum suitability was observed at depths of 855 m for striped dolphins, 845 m for fin whales, 895 m for Cuvier’s beaked whales, and 843 m for sperm whales. The proximity of canyon–like features was an important factor for both the fin whale and the striped dolphin, and exposure to the east was an important factor for both the fin whale and sperm whale. Habitat suitability maps clearly show that the Caprera Canyon provides a large area of highly suitable habitat for all the species analysed. Moreover, habitat suitability models allowed to account for the uneven survey effort in the study area, revealing smaller patches of suitable habitats elsewhere, notably above the canyon–like features south of the Caprera Canyon. Results of the best fitting MaxEnt models for each species were obtained by including only variables with a contribution >5% (calculated as the arithmetic mean between percent contribution and permutation importance). The absence of a value indicates that the variable was not included in the best model (Table 2).

## Discussion

The present study provides data on the distribution, relative abundance, and habitat use of cetaceans inhabiting the Caprera Canyon over a 9–year period. This is the first comprehensive study of the area, supporting the hypothesis that the Caprera Canyon and the surrounding areas can be considered a hotspot of cetacean in the Mediterranean Sea (*sensu* Moulins *et al.* [80]). This area hosts a diverse cetacean community and high cetacean densities, including 7 out of the 8 regularly occurring Mediterranean cetacean species. Notably, the Mediterranean subpopulations of 4 of the species observed in the study area (fin whale, sperm whale, common dolphin, and Risso’s dolphin) have recently been re–assessed and listed as Endangered by the International Union for Conservation of Nature (IUCN) Red list [81–84], underlining the conservation significance of the Caprera Canyon and its broader implications. In addition to highlighting its intrinsic conservation value, the present study enhances the scientific significance of the study area by providing an extensive ecological database, which will be valuable for future comparative analyses and species assessments. Despite the extended study period, multi–year trends were not detected in species’ relative encounter rates. This may result from high variability in encounter rate data, which could mask ongoing trends, or it could reflect actual population stability. Additionally, 9 years may be insufficient to detect trends in such long–lived species. However, the database associated with the present study provides a robust baseline, which in the future will significantly contribute to a better understanding of the long–term cetacean population trends in the Mediterranean.

To streamline the discussion, details on investigated species are also provided in Supporting Information (S2 File), including species–specific sheets, sightings summaries, and insights into distribution, behaviour, habitat use, and conservation concerns. Additionally, these sheets include interesting anecdotal observations and photographic documentation of uncommon or particularly notable sightings.

Overall, the Caprera Canyon stands out among Mediterranean areas where similar studies have been conducted due its high species richness and abundant sightings. For instance, the overall cetacean encounter rate (ER, sightings/100 km) in the Caprera Canyon area (mean ER ± SD = 10.6 ± 7.4) is approximately double that recorded in the Pelagos Sanctuary (ER = 3.9–4.7 sightings/100 km; [80,85]) and in the central Catalan coast (ER = 3.84 sightings/100 km; [10]). It is also significantly higher than the encounter rates reported from the southern Adriatic Sea and south of Sicily (ER = 1.1–0.8 sightings/100 km; [86]). More notably, ERs suggest that certain species are particularly abundant in the study area. A striking example is Cuvier’s beaked whale, which, in the Caprera Canyon, shows one of the highest ER values ever recorded in the Mediterranean Sea (ER = 1.80 sightings/100 km), compared to ER = 0.1–0.6 in the northern Pelagos Sanctuary [87], ER = 1.88 in the central–northern Tyrrhenian Sea [88], and lower values in other Mediterranean areas [89]). Considering species richness and biodiversity, the study area hosts a relatively rich cetacean community, similar to that reported in the central Tyrrhenian Sea (H’ = 0.66; E = 0.27) [17], where the high dominance of striped dolphin lowers the values of diversity indices.

The presence of foetal folds on several observed calves suggests that they were born within or close to the study area. Behavioural observations indicate that the study area serves as both a feeding and breeding ground for most cetacean species. The sighting locations indicate that the distribution of cetaceans in the Caprera Canyon and adjacent waters depends on species–specific habitat requirements, as reflected in the habitat suitability maps of some common species. Based on habitat suitability and observational data, cetacean species are also likely to occur outside the areas surveyed in greater detail (Fig 1). This suggests that the Caprera Canyon may be part of a larger, ecologically important area for cetaceans in the central–western Tyrrhenian Sea. Given the existence of poorly surveyed regions in the Mediterranean Sea [9] and the critical importance of identifying conservation–relevant areas for cetaceans, even on a small scale [11], our study highlights the urgent need to fill the data gaps. Species distribution models that incorporate all these areas could provide valuable insights into the most suitable areas for cetaceans on a larger scale, helping to focus monitoring efforts and conservation strategies. In this regard, future assessments should incorporate oceanographic variables (e.g., remote sensing data on sea level anomalies, surface temperature, and chlorophyll–a concentration) into more advanced species distribution models, such as Generalized Additive Models, to enhance predictive accuracy and address limitations in our current approach, which relies solely on presence–only data and seabed topography. The addition of new data collected in the study area could provide further insights into key aspects of species ecology and population dynamics, including multi–year variability and trends. Additionally, correlating species distribution with potential threats would provide a more comprehensive tool for guiding conservation efforts and ensuring the long–term continuity of high marine biodiversity values in the region.

### Implications for conservation

Although detailed threat assessments are lacking, the Caprera Canyon faces significant risks similar to those affecting cetaceans throughout the Mediterranean. The intense marine traffic in the area [90] heightens the risk of ship strikes involving cetaceans and contributes to environmental contamination (e.g., fuel spills). The canyon is also highly vulnerable to marine litter, with large accumulations of microplastics on the seabed [91], as well as floating and submerged ‘ghost’ fishing gear, posing risks of plastic ingestion and entanglement for cetaceans [92]. Nevertheless, the results of the present study indicate that the area hosts a diverse and apparently well–preserved cetacean community, characterized by relatively high ERs which remained apparently stable over the 9–year study period. However, in the absence of historical reference data, it is difficult to determine to what extent the present situation resembles a hypothetical optimal, pristine state and whether the area could support even greater biodiversity and cetacean abundances. In any case, we suggest that specific conservation measures and actions may be needed to enhance or maintain the conservation status of the area and cetacean populations, and to prevent the further spread of risk factors. As previously mentioned, (see ‘Study Area’), although the Caprera Canyon is adjacent to or borders various national and international marine protected areas, these areas lack specific, legally binding conservation measures for pelagic cetaceans. They primarily encompass waters on the continental shelf and provide solely specific protection for bottlenose dolphins.

To promote cetacean conservation, our study aimed to assess the Caprera Canyon’s eligibility for designation as an Important Marine Mammal Area (IMMA) [39,40]. In 2016, the Caprera Canyon was already proposed as an IMMA candidate based on preliminary data [35,93] and selected as an Area of Interest (AoI) [94], pending quantitative scientific data to further support its candidacy. Our findings likely confirm the Caprera Canyon and adjacent areas meet the criteria outlined for establishing an IMMA: i) it hosts significant concentrations and diversity of marine mammals, including cetaceans and the Vulnerable (IUCN Red List) monk seal *Monachus monachus* (Hermann, 1779) [95], further enhancing its conservation importance; ii) it serves both as feeding and breeding grounds, making it an important habitat for cetacean providing suitable environmental conditions and resources necessary for mating, calving, and foraging; and iii) it is likely crossed by the migratory routes of fin whales [96].

IMMAs are increasingly used in environmental impact assessments, as well as in international, national, and supra–regional conservation policy and management initiatives, including the design and management of Marine Protected Areas (MPAs) and MPA network extensions [40]. We consider the publication of the present study and the hopeful formal designation of the Caprera Canyon as an IMMA, the first steps towards attracting national and international conservation attention to the marine mammals inhabiting the area. Although non–regulatory, its designation will serve as a tool to promote public and stakeholder engagement in marine conservation, potentially leading to effective conservation measures. Considering the wide range of cetaceans and other highly mobile species with dynamic spatial distributions, a pelagic MPA could be established as part of a network of designed MPAs for wide–ranging marine top predators, as suggested by [34]. For example, the Caprera Canyon could represent a core area of the proposed central Tyrrhenian Sea open–sea SPAMI [97].

## Conclusion

Before this study, little was known about the species richness and distribution of pelagic cetaceans off north–eastern coast of Sardinia, particularly in the Caprera Canyon. The present study, based on 810 sightings of 8 species and 8443 km on–effort surveying over 9 years, helps fills this knowledge gap and provides a reference database for future research on the ecology and long–term dynamics of cetaceans in this area, as well as their interactions with impactful human activities. Overall, the high diversity of cetacean recorded off north–eastern Sardinia seems to be related to both habitat diversity and the presence of a high productivity area associated with the canyon system. Although the study area undoubtedly represents only a small fraction of the range of Mediterranean cetacean populations, as well as of the areas covered by most observed individuals, it likely constitutes an important piece of the mosaic of habitats and regions essential for the survival of highly mobile cetaceans in the western Mediterranean Sea.

The high cetacean diversity, the high relative abundance of several species, of which 4 considered endangered to extinction and 1 vulnerable, and the observation of large groups with calves suggest considering the Caprera Canyon as a hotspot of cetaceans of great conservation concern in the Mediterranean Sea, highlighting that the protection of the Caprera Canyon should be prioritized. This study does not quantify the impacts of human activities on cetacean populations, but future research should assess these interactions. The area potentially meets the criteria for Important Marine Mammal Area (IMMA) designation, which would drive effective conservation actions and public engagement. An IMMA designation may also deter harmful human activities, such as intensification of maritime traffic, fishing pressure and pollution. Finally, establishing a Marine Protected Area (MPA) within a network of MPAs for wide–ranging marine predators could be crucial for long–term cetacean conservation in the region.

## Supporting information

S1 Fig Sampling effort map. In red the most surveyed areas.
(PDF)

S2 Fig Response curves of the variables included in the best fitting models. (PDF)

S1 File Supporting material. Survey protocols.
(PDF)

S2 File Supporting material. Sighting summary and ecological profiles of cetaceans in the Caprera Canyon.
(PDF)

S3 File Striped dolphin in the Caprera Canyon. (JPG)
