# Supplementary material for: The Caprera Canyon (north–eastern Sardinia): A hotspot of cetacean diversity in the western Mediterranean Sea
Source: PLoS One. 2025 Jul 9;20(7):e0326426. doi: 10.1371/journal.pone.0326426 (PMC12240396; doi:10.1371/journal.pone.0326426)
Supplement: S1 File — Survey protocols. (PDF) [file pone.0326426.s003.pdf]

Supporting Information S3

Title: The Caprera Canyon (north–eastern Sardinia): a hotspot of cetacean diversity in the western Mediterranean Sea

Authors: Luca Bittau[1*], Renata Manconi[2], Mariliana Leotta[1*], Rossana Tenerelli[1], Mattia Cristina Leone[2], Elena Fontanesi[3], Federica Fonda[4], Ginevra Boldrocchi[5,6], Sandro Carniel[6,7], Rocco Tiberti[8]

[1] SEA ME, Italy; [2] University of Sassari, Italy; [3] Delfini del Ponente APS, Italy; [4] University of Trieste, Italy; [5] University of Insubria, Italy; [6] One Ocean Foundation, Italy; [7] NATO STO Centre for Maritime Research and Experimentation, Italy; [8] University of Calabria, Italy

**Corresponding authors*
E–mail: lucabittau@seame.it (LB)
E–mail: mariliana.leotta@gmail.com (ML)

Survey protocols

Data collection methods

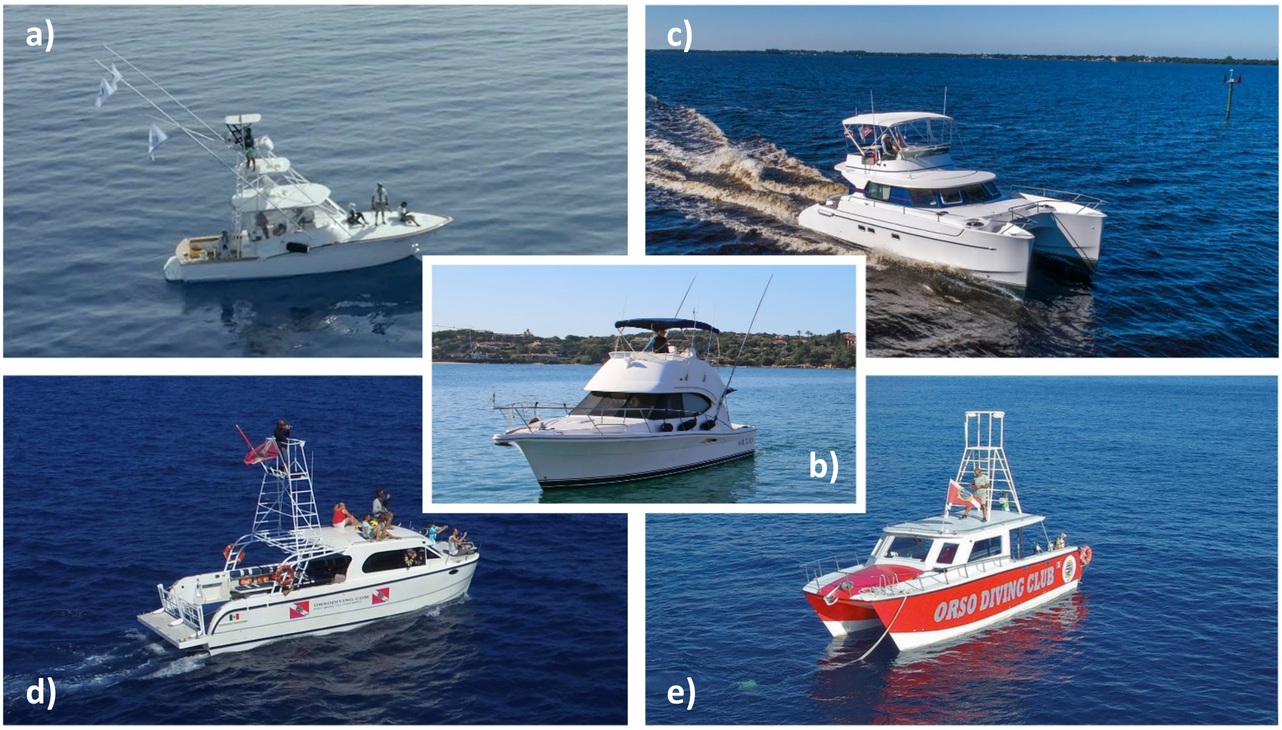

Fig. S3 1. The five boats used for cetacean surveys during the present study: a) the 9 m monohull motorboat (*Strike*); b) the 10 m monohull motorboat (*Touche*); and the 11–m power catamarans: c) *Freedom Cat,* d) *Orso Cat* and e) *Scuba Cat*, the last two operated by Whale Watching Sardinia. All boats were equipped with an observation platform (fly–bridge or observation tower).

Visual boat surveys were conducted on single days along line transects, with the aim of detecting as many cetacean species as possible. The observation team consisted of at least 3 members: starboard, port, and central observers including at least 2 experienced observers proficient in visual monitoring and data collection of cetaceans. Observers were trained using a consistent protocol, with less experienced individuals paired with experienced ones to minimize potential biases in data collection.

Observers conducted 360° scans by naked eye from the observation platform, occasionally aided by 7x50 compass binoculars. Starboard and port observers performed a visual scan at 180° both sides of the vessel track, up to 2 km from the transect line, recording all survey and sighting data, while the central observer at 360° out to the horizon.

Survey data were recorded either on a standard data sheet or with an audio recorder and later entered into a Microsoft Excel datasheet upon returning to port. Navigation data and sighting positions were logged using Global Positioning System (GPS) equipment, including Garmin 64S, Garmin E–Trex 20, Garmin E–Trex–Venture, and the onboard GPS/Plotter systems Garmin 540S and Garmin 1020XS. The vessels position, navigation speed (in knots), and direction (in degrees) were logged every 5 seconds from departure until arrival at port.

Throughout the survey, sea and weather conditions were also recorded every hour, and whenever a significant change was observed. Environmental data comprised wind direction (in degrees), wind intensity (measured on the Beaufort scale), sea state (according to the Douglas scale), swell height (approximated in cm), visibility (in km), cloud cover (expressed as a percentage, and presence of rain, adapted from [1]). If rain was forecasted, we postponed the survey date. If it rained while we were already in the field, we ceased effort.

The survey protocol included 2 effort modes: 'on–effort' and 'off–effort'. The cetacean survey was considered on–effort, indicating active cetacean searching, under the following conditions: (i) vessel speed between 6 and 10 knots; (ii) at least 2 experienced observers at their designated positions; (iii) favourable environmental conditions, including full daylight (within ±30 minutes from sunrise/sunset), moderate wind speed (Beaufort scale ≤3), calm sea (Douglas scale ≤2), moderate swell (≤50 cm), good visibility (≥5 km) and no rain. Environmental conditions can indeed affect the probability of detecting cetaceans [2–5], especially for smaller dolphins [6,7] and elusive species like the Cuvier's beaked whale (*Ziphius cavirostris*) [8]. The on–effort starting point of each survey was logged at a randomly established point along the transect, if the conditions listed above were met. The on–effort mode was suspended, thereby transitioning to the off–effort mode, whenever a cetacean was sighted or at least one of the conditions listed above was not met, such as in the presence of unsuitable environmental conditions. The on–effort mode was eventually resumed, for example, at the end of the sighting or as soon as the environmental conditions became suitable again, and definitively interrupted when the decision was made to return quickly to the port. The start and end times/points of the on–effort tracks were accurately recorded along the routes. Consequently, the overall on–effort part of a survey was the sum of all the on–effort sections of the boat track–line recorded during each survey.

Sighting protocol

Whenever a cetacean was sighted, the on–effort mode was suspended, to enable the adoption of the sighting protocol. A "sighting" is defined as the positive detection of cetaceans, involving the observation of either a solitary individual or a group of individuals of the same species seen simultaneously at the same location. For delphinids and beaked whales, individuals were considered part of the same group if observed in apparent association, typically moving in the same

direction (considering the entire sighting) and often, but not always, involved in the same activity, such as coordinated surfacing and diving patterns (modified from [9,10]) within a distance of less than 0.5 km from each other (modified from [11]). Large cetaceans were considered a group if one or more individuals remained together and swam in a coordinated manner throughout the sighting, within a distance of 1.0 km for fin whales (modified from [11]). For sperm whale groups, we adopted the definitions of an "aggregation group" (solitary male, male aggregation, social unit, and unclassified), according to [12]. The sighting protocol involved a series of steps for data collection. First, the initial position of the single animal (or group) was determined by estimating the sighting distance and bearing angle from the vessel. Distance was estimated by converting the reticule scale in binoculars into kilometric distance [13], while the bearing angle was determined using the compass integrated into the binoculars. Next, the vessel approached the animals to collect additional sighting details, recording the primary position (i.e., the vessel's position at the minimum distance from the animals during the initial approach) and accurately documenting all sighting data. These primary sighting positions were used to unambiguously define the sighting occurrence. This procedure resembles the "closing mode", as described by [14] and [15], where observers interrupt their normal searching procedures upon sighting a group of cetaceans (transitioning to 'off–effort'). The vessel then leaves the transect line and approaches the group to enable species identification and reliable group size estimation.

If cetaceans dive before the vessel can approach them and are no longer spotted, but it was still possible to perform species identification and group size estimates at a distance, the vessel then reaches the dive position to record the primary sighting point (modified from [2]). In a few cases (n = 66; 5.9% of the total sightings), approaching the cetaceans was not feasible, such as when animals disappeared from sight, but species identification and group size estimates were successfully recorded at a distance. In such cases, the primary position was determined using range and bearing estimation with reticule–compass binoculars. Only the sightings with fully reliable species identification were recorded during the present study. Sighting data include initial distance and bearing angle from the vessel, primary position, end–sighting position (when the animals were left), sighting start and end times, species identification, group size, age class (when possible), presence/absence of calves, recording of focal group behavioural data [16] and photo–identification data, the latter available for future studies. Group size (i.e., the estimated total number of individuals in a group) was obtained in the field by independent counts performed by at least 2 experienced observers [17]. Each observer repeatedly counted the groups, providing minimum, maximum, and best group size estimates. The final estimate was then calculated as the mean best estimate of all observers. Photo–identification data was also used to confirm/correct the group size estimates, except for schools of striped dolphin and short–beaked common dolphin.

At the end of the sighting, the on–effort mode was resumed, and the vessel re–joined the projected track–line. To avoid a stop/start effect after each sighting [18,19] and potential double counting of previous sightings, the trackline was rejoined by following a convergent route.

Thus, it was avoided to return to the point where the vessel left the transect for a sighting (according to [20]), and the survey continued from the sighting point along a new trajectory convergent to the original trackline. Our survey protocol adheres to widely accepted codes of conduct to minimize disturbance to the observed animals, following the guidelines established by the Pelagos Sanctuary and the ACCOBAMS Agreement.

References

1. Kiszka J, Macleod K, Van Canneyt O, Walker D, Ridoux V. Distribution, encounter rates, and habitat characteristics of toothed cetaceans in the Bay of Biscay and adjacent waters from platform-of-opportunity data. ICES Journal of Marine Science. 2007;64: 1033–1043.

2. Barlow J, Gerrodette T, Forcada J. Factors affecting perpendicular sighting distances on shipboard line-transect surveys for cetaceans. Journal of Cetacean Research and Management. 2001; 201–212.

3. Redfern J V, Barlow J, Ballance LT, Gerrodette T, Becker EA. Absence of scale dependence in dolphin–habitat models for the eastern tropical Pacific Ocean. Mar Ecol Prog Ser. 2008;363: 1–14.

4. Bailey H, Corkrey R, Cheney B, Thompson PM. Analyzing temporally correlated dolphin sightings data using generalized estimating equations. Mar Mamm Sci. 2013;29: 123–141. doi:https://doi.org/10.1111/j.1748-7692.2011.00552.x

5. Thiele D, Chester ET, Gill PC. Cetacean distribution off Eastern Antarctica (80–150 E) during the Austral summer of 1995/1996. Deep Sea Research Part II: Topical Studies in Oceanography. 2000;47: 2543–2572.

6. Hammond PS, Benke H, Breggren P, Collet A, Heide-Jørgensen MP, Heimlich-Boran S, et al. The distribution and abundance of harbour porpoises and other small cetaceans in the North Sea and adjacent waters. Life 92–2/UK/027. ICES; 1995. p. 239.

7. Gannier A. Summer distribution and relative abundance of delphinids in the Mediterranean Sea. Revue d’Ecologie, Terre et Vie. 2005;60: 223–238.

8. Gannier A, Epinat J. Cuvier’s beaked whale distribution in the Mediterranean Sea: results from small boat surveys 1996–2007. Journal of the Marine Biological Association of the United Kingdom. 2008/03/17. 2008;88: 1245–1251. doi:DOI: 10.1017/S0025315408000428

9. Shane SH. Behavior and ecology of the bottlenose dolphin at Sanibel Island, Florida. The bottlenose dolphin. 1990; 245–265.

10. Bearzi G, Politi E, Agazzi S, Bruno S, Costa M, Bonizzoni S. Occurrence and present status of coastal dolphins (Delphinus delphis and Tursiops truncatus) in the eastern Ionian Sea. Aquat Conserv. 2005;15: 243–257.

11. Azzellino A, Gaspari S, Airoldi S, Nani B. Habitat use and preferences of cetaceans along the continental slope and adjacent waters in the western Ligurian Sea. Deep Sea Research Part I: Oceanographic Research Papers. 2008;55: 296–323. doi:10.1016/j.dsr.2007.11.006

12. Frantzis A, Alexiadou P, Gkikopoulou KC. Sperm whale occurrence, site fidelity and population structure along the Hellenic Trench (Greece, Mediterranean Sea). Aquat Conserv. 2014;24: 83–102. doi:https://doi.org/10.1002/aqc.2435

13. Kinzey D, Gerrodette T. Distance measurements using binoculars from ships at sea: Accuracy, precision and effects of refraction. J Cetacean Res Manag. 2003;5: 159–171.

14. Dawson S, Wade P, Slooten E, Barlow JAY. Design and field methods for sighting surveys of cetaceans in coastal and riverine habitats. 2008.

15. Schwarz LK, Gerrodette T, Archer FI. Comparison of closing and passing mode from a line-transect survey of delphinids in the eastern Tropical Pacific Ocean. J Cetacean Res Manage. 2010;11: 253–265.

16. Mann J. Behavioral sampling methods for cetaceans: a review and critique. Mar Mamm Sci. 1999;15: 102–122. doi:https://doi.org/10.1111/j.1748-7692.1999.tb00784.x

17. Gerrodette T, Perryman WL, Oedekoven CS. Accuracy and precision of dolphin group size estimates. Mar Mamm Sci. 2019;35: 22–39. doi:https://doi.org/10.1111/mms.12506

18. Kishino H, Kasamatsu F. Comparison of closing and passing mode procedures used in sighting surveys. Report of the International Whaling Commission. 1987;37: 253–258.

19. Hiby AR. Survey techniques for estimating abundance of cetaceans. Rep int Whal Commn (special issue). 1989;11: 45–80.

20. Forcada J, Aguilar A, Hammond PS, Pastor X, Aguilar R. Distribution and numbers of striped dolphins in the western Mediterranean Sea after the 1990 epizootic outbreak. Mar Mamm Sci. 1994;10: 137–150.
