# Supplementary material for: The Caprera Canyon (north–eastern Sardinia): A hotspot of cetacean diversity in the western Mediterranean Sea
Source: PLoS One. 2025 Jul 9;20(7):e0326426. doi: 10.1371/journal.pone.0326426 (PMC12240396; doi:10.1371/journal.pone.0326426)
Supplement: S2 File — Sighting summary and ecological profiles of cetaceans in the Caprera Canyon. (PDF) [file pone.0326426.s004.pdf]

Supporting Information S4

Title: The Caprera Canyon (north–eastern Sardinia): a hotspot of cetacean diversity in the western Mediterranean Sea

Authors: Luca Bittau[1*], Renata Manconi[2], Mariliana Leotta[1*], Rossana Tenerelli[1], Mattia Cristina Leone[2], Elena Fontanesi[3], Federica Fonda[4], Ginevra Boldrocchi[5,6], Sandro Carniel[6,7], Rocco Tiberti[8]

[1] *SEA ME, Italy;*
[2] *University of Sassari, Italy;*
[3] *Delfini del Ponente APS, Italy;*
[4] *University of Trieste, Italy;*
[5] *University of Insubria, Italy;*
[6] *One Ocean Foundation, Italy;*
[7] *NATO STO Centre for Maritime Research and Experimentation, Italy;*
[8] *University of Calabria, Italy*

Sighting summary and ecological profiles of cetaceans in the Caprera Canyon.

A detailed summary of the investigated species is provided here, including species–specific sheets, sightings summaries, and insights into distribution, behaviour, habitat use, and conservation concerns. These sheets also include interesting anecdotal observations and photographic documentation of uncommon or particularly notable sightings. The detectability of cetaceans at sea and the ability to identify them at the species level depend on the survey protocol used [1,2]. For instance, identifying small dolphin species, such as the striped dolphin and common dolphin, during aerial surveys can be challenging [3]. To ensure consistency, the results of this study were compared with those from studies employing similar protocols—specifically, boat–based surveys conducted in other Mediterranean areas using comparable descriptor parameters (e.g., encounter rates calculated based on linear distance, group size, and diversity indices). For example, encounter rates were compared by counting the number of visual sightings (single individuals or groups) along the linear distance travelled on–effort by similar vessels under favourable sea and weather conditions.

Between 2011 and 2019, a total of 216 one-day boat surveys were conducted in the Caprera Canyon and surrounding areas, covering a total of 29175 km (20732 km off-effort and 8443 km on-effort). A total of 1110 cetacean sightings (810 on-effort) were recorded, representing 11867 individuals (8747 observed on-effort). Sightings with incomplete data or uncertain species identification were excluded.

Sighting summary and ecological profiles of cetaceans in the Caprera Canyon.

Striped dolphin *Stenella coeruleoalba*

Sightings summary:

Table S4.1 Sighting summary.

Year	No. Sightings on/off effort	No. individuals on/off effort	Group size mean ± SD	ER (No. / 100 km) mean ± SD	Depth (m) mean ± SD	Slope (%) mean ± SD
2011	83/99	1059/1285	13 ± 10.9	7.7 ± 5.5	786 ± 155	5.5 ± 4.9
2012	83/105	1459/1799	17.1 ± 14.1	7 ± 4.3	815 ± 160	5.7 ± 5.1
2013	55/73	1526/1813	24.8 ± 19.1	4.7 ± 3.7	776 ± 136	3.6 ± 4
2014	44/67	768/1197	17.9 ± 12.3	4.8 ± 6	800 ± 138	4.8 ± 4.9
2015	39/64	710/1023	16 ± 12	4.5 ± 3.8	788 ± 161	5.6 ± 5
2016	46/65	889/1164	17.9 ± 14.6	6.9 ± 6.4	806 ± 141	5.8 ± 5.3
2017	47/65	730/946	14.6 ± 11.8	4.4 ± 4	797 ± 136	6.6 ± 5.5
2018	18/24	242/404	16.8 ± 14.7	5.5 ± 2.4	710 ± 154	5.9 ± 5.6
2019	32/42	602/801	19.1 ± 15.8	3.6 ± 3	807 ± 140	3.9 ± 4.1
**TOT.:**	**447/604**	**7985/10432**	**17.3 ± 14.2**	**5.4 ± 4.8**	**793 ± 149**	**5.3 ± 5**

ER: Encounter Rate as number of sightings per 100 km.

Sighting dynamics: The striped dolphin was regularly recorded throughout the study period and surveys, suggesting that this is a resident species as observed in other Mediterranean areas [4] . No clear multiannual trends were observed for this species.

Frequency of occurrence and relative abundance: Similarly to what reported in other studies focusing on the western Mediterranean Sea (e.g.,[1,4–9], the striped dolphin was the most abundant cetacean species in the Caprera Canyon. This species exhibited particularly high relative abundance, with encounter rate values (ER = 0.80; n = 27; Table 1) exceeding those recorded for the central Tyrrhenian Sea [10].

Group size: Striped dolphin's group size (GS) and group size range (GSR) in the study area were similar to or slightly lower than what observed in other studies [1,5,11]. For example, average GS of 18.06 and GSR = 3–100 was observed in the central–western Mediterranean Sea, including waters off eastern Sardinia and the eastern Pelagos Sanctuary [5]. In contrast, higher mean group sizes were estimated in several regions of the Mediterranean Sea (average GS = 28.3) and the northern Tyrrhenian Sea (GS = 37.4) [1].

Distribution and habitat preference: Concerning habitat use and preference, the striped dolphin is mainly associated to the lower slope (mean depth 824 m) in offshore waters and canyon–like features (Fig 6; Table 2). Literature data report similar depth distributions, such as mean depth of 1622 m (range 189–3886 m) in the Mediterranean Sea and contiguous Atlantic area [11], a mean depth of around 1000 m in the central Tyrrhenian Sea [6], and a mean depth of 1490 m (range 25–2500 m) in the central Mediterranean Sea [9]. Preference for waters deeper than 600 m deep was recorded off southern Spain [12], and a mean depth of 1759 m was recorded in 7 regions of the Mediterranean Sea [1]. In the present study, the distribution of striped dolphin partially overlaps the areas where other species occurred, except for the bottlenose dolphin, which was primarily restricted to the continental shelf. According to the observed behaviours and group composition, striped dolphins use the Caprera Canyon and adjacent waters both as a feeding and breeding area. Groups of striped dolphins usually included juveniles and calves, with the latter in 30.1% of the overall sightings.

Behavioural observations: On a few occasions, striped dolphins were observed in association with fin whales (n = 4) and common dolphins (n = 3). On 17 August 2013, a female striped dolphin was recorded in close association with a few–hour old newborn (photographic documentation in Fig S4 1a, 1b), identified according to the presence of foetal folds, a curled tail, small whiskers on the rostrum, and blood near the mouth, clumsy swimming close to the putative mother; in addition, the female lifting the newborn at the surface with the head was clearly and repeatedly observed.

Threats and conservation: The striped dolphin is listed as Least Concern in the IUCN Red List of threatened species [13].

Other observations: Given that the high dominance of a single species has a suppression effect on some biodiversity indices, the large number of sightings of striped dolphin obtained in this study may explain why the Shannon index in the Caprera Canyon was lower than the value (H' = 1.42) recorded in the northern Tyrrhenian Sea, where the striped dolphin was less dominant, but had a lower sighting frequency (62.5%) [1].

Relevant photographic documentation. Fig S4 1a, 1b. Striped dolphin few hours old newborn, photographed on 7 August 2013.

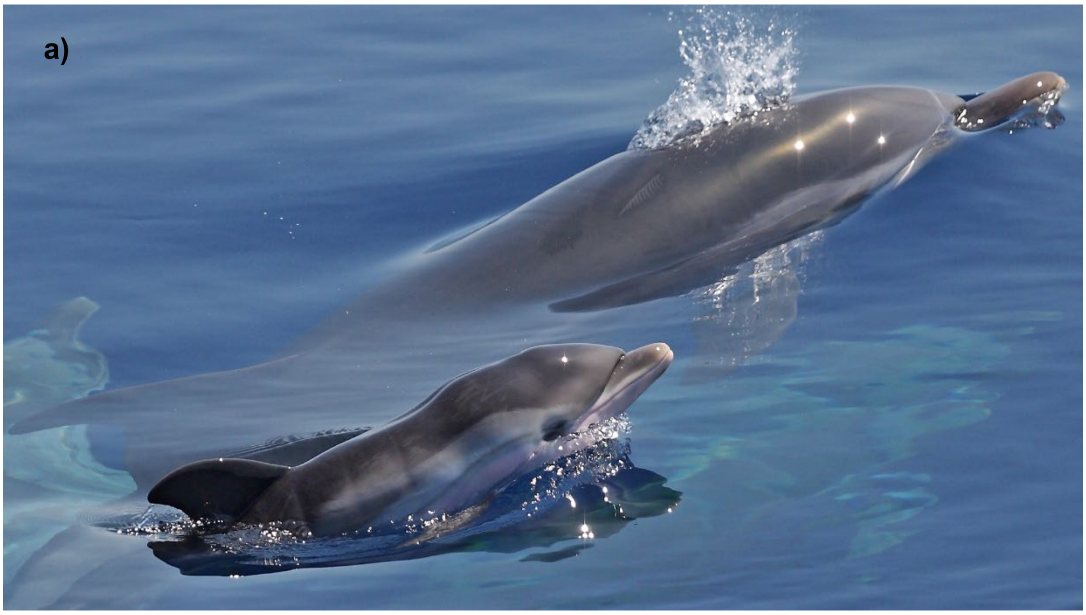

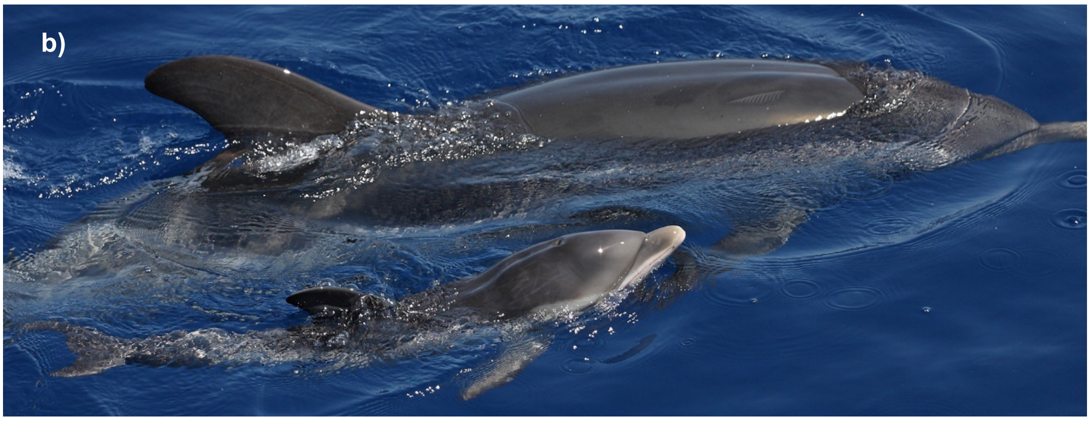

Fin whale *Balaenoptera physalus*

Sightings summary:

Table S4.2 Sighting summary.

Year	No. Sightings on/off effort	No. individuals on/off effort	Group size mean ± SD	ER mean ± SD	Depth (m) mean ± SD	Slope (%) mean ± SD
2011	40/44	63/68	1.6 ± 0.7	4.1 ± 3.8	767 ± 278	5.9 ± 5
2012	22/25	37/42	1.7 ± 0.8	2.5 ± 3.2	756 ± 210	3.4 ± 2.6
2013	37/46	60/70	1.5 ± 0.9	3 ± 3.1	802 ± 115	5.2 ± 5.8
2014	39/47	67/80	1.7 ± 0.8	5.1 ± 5.8	845 ± 150	4.9 ± 4.1
2015	17/20	26/32	1.6 ± 0.7	2.9 ± 5	877 ± 98	6.4 ± 5.9
2016	15/16	27/28	1.8 ± 0.9	2.5 ± 2.8	947 ± 107	7.5 ± 7.4
2017	19/23	29/35	1.5 ± 0.8	2.1 ± 4.8	810 ± 209	5.6 ± 5.4
2018	14/20	22/31	1.6 ± 0.8	5.1 ± 5.9	754 ± 228	6.2 ± 4.8
2019	3/3	3/3	1 ± 0	0.4 ± 1.1	832 ± 6	2 ± 1.7
**TOT.:**	**206/244**	**334/389**	**1.6 ± 0.8**	**3 ± 4.2**	**812 ± 192**	**5.4 ± 5.1**

ER: Encounter Rate as number of sightings per 100 km.

Sighting dynamics: The fin whale was regularly recorded throughout the study period. No clear multiannual trends were observed for this species.

Frequency of occurrence and relative abundance: It was the second most abundant cetacean species in the study area, showing notably high encounter rates (ER = 3.0) compared to those observed in the central Tyrrhenian Sea from a fixed line–transect (ER = 1.04, n = 72) [14]. Interestingly, the ER recorded in this study is comparable to the highest encounter rate estimated in 2012 during a 5–year study (2009–2013) in a highly productive area of the north–western Mediterranean Sea, within the Pelagos Sanctuary (ER = 2.34, SD = 2.80, n = 66) [15].

Group size: The estimated group size and group size range obtained from the overall dataset (n = 206) in the Caprera Canyon (mean GS = 1.62; GSR = 1–5; Table 1) were consistent with the average GS = 1.74 recorded between 1990 and 1999 in the Pelagos Sanctuary (n = 540) [16]. Consistent with literature data, in the Caprera Canyon, fin whales were commonly observed either alone (54.9% of sightings) or in pairs (32%), with single individuals or pairs accounting for 86.9% of the overall dataset, while small groups of 3 or more individuals constituted 13.1%. For instance, studies reported that groups of 3 or more individuals represented only 19.1% and 7.0% of the sightings, respectively, in the Pelagos Sanctuary and 3 different subareas in the central–western Mediterranean Sea (including the Pelagos Sanctuary, western, and south–eastern areas) [16,17]. In total, 4 sightings consisted of female–calf pairs, in very close association. The estimated size of the calves ranged from half to around 2/3 of the adult size.

Distribution and habitat preference: Fin whales occur mainly, although not exclusively, in offshore temperate waters [18], although their occurrence over the continental shelf is not unusual [9]. Accordingly, in the present study, most fin whales were recorded above the lower slope waters, but a few sightings were recorded in the outer shelf and shelf–break, off northeastern Sardinia (Fig 5). The occurrence of fin whales in the continental shelf waters and the observation of feeding behaviour both in shallow and deep waters, support the idea that fin whale may use the Caprera Canyon and adjacent waters as a feeding habitat. The high encounter rates and group size suggest that fin whales may gather in large groups in this area off north–eastern Sardinia.

At a small scale, such as the Caprera Canyon, a feeding area of fin whales may vary a lot between years, depending on temporary environmental variables (e.g., SST and chl a, as described by [19]. However, focusing on an annual resolution monitoring could lead to imprecise results, due to the heterogeneity of patterns at the monthly scale [15]. Indeed, the Mediterranean fin whale is thought to be a nomadic

opportunistic species, adapted to exploit localised mesoscale hotspots of productivity that are highly variable in space and time [20,21]. Five fin whales were tagged with satellite transmitters in the strait of Sicily to evaluate movements and habitat use of the species in the western Mediterranean Sea [22]. One of the whales tagged in 2015 undertook long–distance movement from the southern Strait of Sicily to the northern Ligurian Sea, both potential feeding habitats for the fin whale. During this clearly observed early– to mid–spring latitudinal movement, the fin whale crossed the study area through the Caprera Canyon waters. While the sample size is limited, this data, along with the observed travelling behaviour, could support the hypothesis that the study area serves as a potential route for fin whales during their seasonal movements across the Mediterranean.

Behavioural observations: Feeding, resting, travelling, nursing, and breaching were the observed behaviours of fin whales during the study period. In late spring and summer, some individuals and groups were observed repeatedly diving and surfacing in the same area, presumably engaged in feeding activities, as evidenced by several episodes of defecation recorded throughout the study period. Furthermore, in 2011 and 2012, single individuals or pairs were observed displaying feeding behaviour in the shallow continental shelf waters (90–100 m deep), approximately 5 miles away from the Caprera Canyon head. In addition, several sightings of fin whale groups were recorded during spring and autumn, with the whales clearly exhibiting northward or southward "travelling" behaviour, respectively.

Threats and conservation: The mediterranean subpopulation of fin whale is classified as Endangered in the IUCN Red List of threatened species [23].

Other observations: Several individuals (n = 7) were observed with large wounds, such as propeller scars, and were considered as victims of a boat collision. Types of injuries were propeller cuts on the back, behind the dorsal fin, and on the fluke. At least 2 near–miss collisions and a non–lethal episode of collision between an individual and a small vessel have been observed (Fig S4. Furthermore, another collision episode was documented between a fin whale and a small vessel near the eastern coast of Sardinia (Bittau, personal communication). See Fig

Relevant photographic documentation: Fig S4 2a, 2b. Propeller wounds on fin whales.

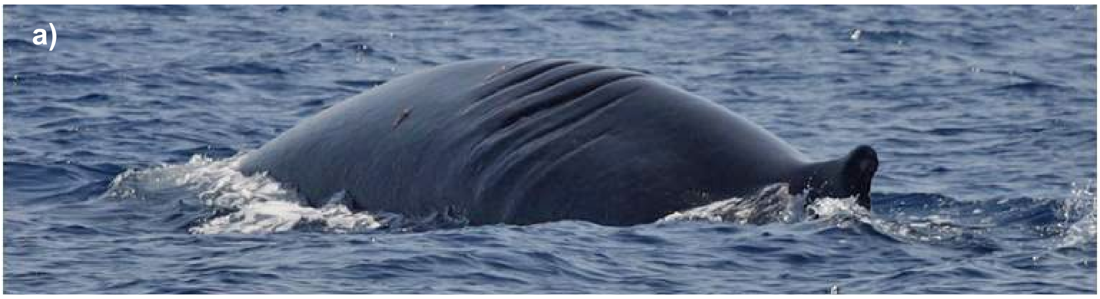

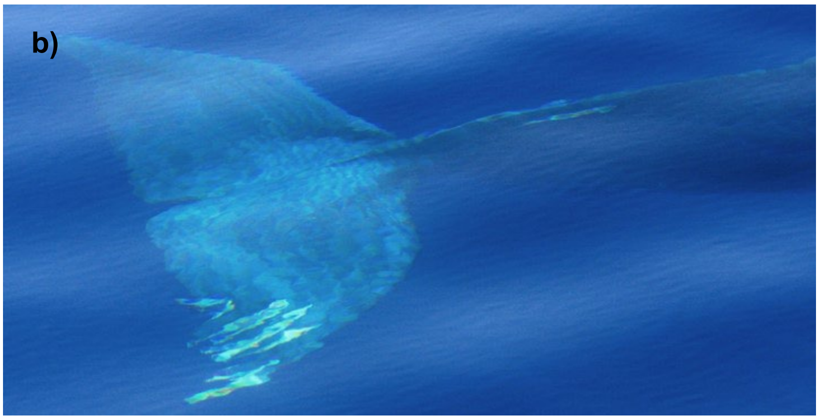

Cuvier's beaked whale *Ziphius cavirostris*

Sightings summary:

Table S4.3 Sighting summary.

No. Sightings	No. individuals	Group size	ER	Depth (m)	Slope (%)
on/off effort	on/off effort	mean ± SD	mean ± SD	mean ± SD	mean ± SD
9/9	21/21	2.3 ± 0.9	1.1 ± 1.9	-842 ± 89	6.6 ± 3.8
21/22	37/38	1.7 ± 0.8	2.1 ± 2.6	-900 ± 102	6.8 ± 5.1
29/33	72/81	2.5 ± 1	2.4 ± 3.1	-854 ± 67	3.5 ± 3.4
14/18	31/42	2.3 ± 1.4	1.6 ± 2	-866 ± 94	6.9 ± 5.2
12/13	27/29	2.2 ± 1.4	1.4 ± 2.2	-846 ± 77	3.5 ± 3.9
19/22	48/54	2.5 ± 2.1	3.8 ± 7	-868 ± 112	6.3 ± 7.4
15/19	31/39	2.1 ± 0.8	1.4 ± 1.6	-895 ± 83	7.9 ± 7.5
1/1	4/4	4 ± 0	0.3 ± 0.9		
10/12	21/25	2.1 ± 0.8	1.2 ± 1.6	-830 ± 70	3.9 ± 2.7
130/149	292/333	2.2 ± 1.2	1.8 ± 3.1	-866 ± 89	5.5 ± 5.3

ER: Encounter Rate as number of sightings per 100 km.

Sighting dynamics: The Cuvier's beaked whale was regularly recorded throughout the study period and surveys, suggesting that this is a resident species as observed in few other Mediterranean areas [24,25]. No clear multiannual trends were observed for this species.

Frequency of occurrence and relative abundance: In this study, the species was the third most sighted in the Caprera Canyon and adjacent waters. The encounter rate (ER = 1.80 sightings/100 km) reported from this canyon is among the highest ever recorded in the Mediterranean Sea, highlighting the importance of this area for the species. In the northern Pelagos Sanctuary, [26] reported encounter rates ranging 0.1–0.6 sightings/100 km and lower values (ER = 0.02–0.081 sightings/100 km) were reported in the central Tyrrhenian Sea [8,27]. However, the latter low encounter rates were obtained using fixed line transect from opportunistic ferry–based surveys and may be due to the long dive behaviour of the species, which, associated to the high vessel's speed can produce lower ER estimates [6]. Conversely, an ER = 1.88 sightings/100 km, value similar to what recorded in the Caprera Canyon, was reported by [28] in the central–northern Tyrrhenian Sea (n = 19, survey effort = 907 km). The high values of encounter rate of this cryptic species, observed with calves over the study period, suggests the Caprera Canyon and adjacent waters as a likely important feeding and breeding area for the Cuvier's beaked whale.

Group size: The mean group size (GS = 2.23) was similar to the average group size recorded in the northern Pelagos Sanctuary (GS = 2.3; [26] and in the central Tyrrhenian Sea (GS = 2.5; [28]), and higher than those recorded in 2007–2011 by [27] in the whole central Tyrrhenian Sea (GS = 1.75 in 1990–1992, and GS = 1.88). 29.5% of sightings were observations of a single individual, while most sightings were of groups of 2 (36.9%), 3 (20.1%) and 13.4% were composed of 4 or more animals. The largest group of Cuvier's beaked whales (8 individuals) was recorded in 2016, which comprised adults, juveniles, and calves. Calves were observed in 18% of the overall sightings of Cuvier's beaked whales. During the study period, calves with foetal folds were observed along with several sightings of female/calf pairs remaining in the area for at least 30 days (Bittau, unpublished photo–identification data).

Distribution and habitat preference: The Cuvier's beaked whale is a cryptic species which is localized in suitable areas and appears to be associated with submarine canyon habitats and continental slopes

[12,26,29–32]. In the northern Pelagos Sanctuary, sightings of this species are mainly concentrated in the hotspot of Genoa Canyon [33]. The central Tyrrhenian Sea was argued to be a favourable habitat for the species [28], in addition to the key areas listed by [34]. A multispecies study carried out with an extensive collective dataset of cetaceans in the Mediterranean Sea showed that the Cuvier's beaked whale is mainly observed in a few relatively small areas, including the Caprera Canyon [35]. Also, habitat use and preference was in line with literature data [27], with the totality of the sightings occurring in an area with high bathymetric variability. Although the sampling was carried out in an area at depths ≤1300 m, results of the present study clearly highlighted that this elusive species mainly occurs at depths <1000 m (91.3 % of the total sightings), with a mean depth of 866 m (Table 1). Similarly, a mean depth of 1000 m was obtained [27], whereas another study [36] also confirmed a preference of the Cuvier's beaked whale for areas around 1000–1500 m depth in the central Tyrrhenian Sea. Results of the present work confirm and reinforce the hypothesis regarding the Caprera Canyon as a "beaked whale hotspot" [37].

Behavioural observations: Logging, travelling, nursing, spy-hopping, tail-slapping and breaching were the observed behaviours of the species. Cuvier's beaked whale was observed in association with fin whales (n = 1) and Sowerby's beaked whale (n = 1).

Threats and conservation: The Mediterranean subpopulation of Cuvier's beaked whale is listed as Vulnerable in the IUCN Red List of threatened species [38].

Other observations:

Relevant photographic documentation: Fig S4 3. A female-calf pair of Cuvier's beaked whales observed in the study area. The calf is still showing foetal folds.

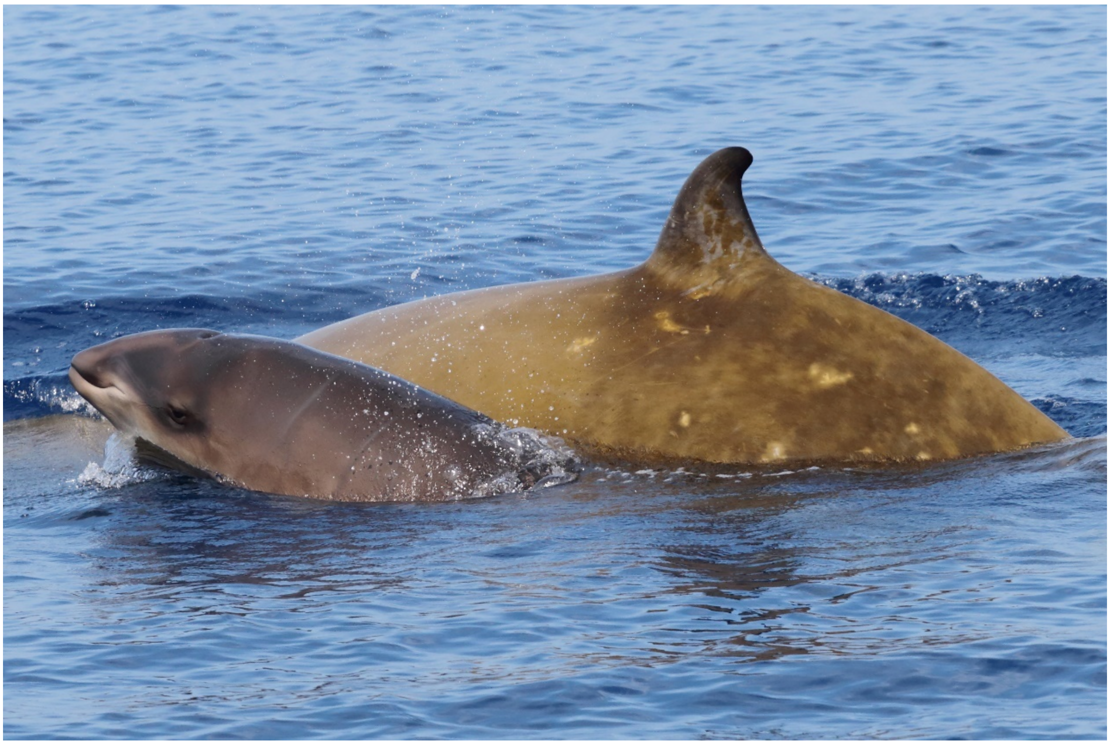

Sperm whale *Physeter macrocephalus*

Sightings summary:

Table S4.4 Sighting summary.

**No. Sightings** on/off effort	**No. individuals** on/off effort	**Group size** mean ± SD	**ER** mean ± SD	**Depth (m)** mean ± SD	**Slope (%)** mean ± SD
0/0	0/0				
1/1	3/3		0.1 ± 0.5		
8/9	27/28	3.1 ± 4.2	0.7 ± 1.2	-776 ± 109	4.3 ± 4.1
1/1	1/1	1 ± 0	0.1 ± 0.5		
1/1	4/4	4 ± 0	0.2 ± 0.8		
0/1	0/2	2 ± 0			
0/0	0/0				
1/1	1/1	1 ± 0	0.3 ± 0.9		
1/1	1/1	1 ± 0	0.1 ± 0.6		
13/15	37/40	2.7 ± 3.3	0.2 ± 0.7	-764 ± 103	5.6 ± 5.1

ER: Encounter Rate as number of sightings per 100 km.

Sighting dynamics: The relatively small number of sightings of sperm whale in the Caprera Canyon area suggests the species is not frequent, albeit the occurrence of 2 sightings of social units. The observation of a surface nursing episode (photographic documentation in Fig S4 4), the close association of calves (<6 m) with adults, and the simultaneous presence of several individuals within a radius of less than 500 m allowed us to confirm the sighting of social units. Multiannual trends were not observed for the species.

Frequency of occurrence and relative abundance: Encounter rate of sperm whale in the Caprera Canyon (ER = 0.17) was similar to the highest ER obtained in the central–northern Tyrrhenian Sea (ER = 0.1, n = 2) [10], and higher than what reported for the south–western Mediterranean basin (ER = 0.037; n = 1) [11]. Although sperm whales in the area were not regularly recorded, it is important to point out that no acoustic monitoring was performed during the study. Indeed, carrying out visual instead of acoustic surveys can result in an underestimation of the relative abundance of sperm whale [39,40]. Nonetheless, the observation of solitary individuals (66.7% of the total sightings) and social units suggested that the area is periodically visited by this species, with herds made up of males, females, calves, and young individuals. Altogether, these observations seem to confirm that sperm whales may use the Caprera Canyon and adjacent waters along their movements though the Mediterranean Sea.

Group size: The sperm whale showed a wide overall group size range (GSR = 1–11). The occurrence of 2 sightings of social units (n = 10 and n = 11) explains the large overall group size range obtained. However, when sightings of social units were excluded from the count, the mean group size was 1.46 ± 0.97 (GSR = 1–4). Solitary individuals, bachelor groups, isolated adult–calf pairs and larger groups (all categories, n = 1) were observed. In the Hellenic Trench, off southern and south–eastern coasts of Greece eastern Mediterranean Sea [41], with a larger dataset obtained similar group size estimates for social units (GS = 8.21; GSR = 4–13; n = 42) and higher for males (GS = 2.47; GSR = 1–5; n = 59). Off Ischia Island (central–eastern Tyrrhenian Sea) a similar overall group size range and a lower group size for social units were reported (GSR = 1–8, n = 99; GSSU = 6.66, n = 6) [42].

Distribution and habitat preference: The occurrence of social units made up of females and calves, along with other sightings of social units off eastern Sardinia (Bittau, pers. comm.) pointed out that the central Tyrrhenian Sea could be a breeding area in the central–western Mediterranean Sea. This

hypothesis requires further dedicated investigations, to better understand how the sperm whales exploit this area, both spatially and temporally.

Behavioural observations: Logging, travelling, nursing, spy-hopping, socializing, and breaching were the observed behaviours of the sperm whales, recorded during the study period.

Threats and conservation: The Mediterranean subpopulation of sperm whales was assessed as Endangered in the IUCN Red List of threatened species [43]. A stranding episode was reported in the area on 28 March 2019 in Porto Cervo (north–eastern coast of Sardinia). It involved an 8-meter-long pregnant sperm whale found dead, with 22 kg of plastic debris and marine litter in its stomach contents [44].

Other observations: During the study period, a sperm whale with atypical white-yellowish colouration was observed in the study area [45]. It can be ascribed either to a form of leucism or albinism, which is an atypical for sperm whales. Photo-identification from the 2015 sighting revealed that this individual had been previously sighted off eastern Sardinia in 2006, near the Tavolara Canyon [45].

Relevant photographic documentation: Fig S4 4. A female sperm whale nursing a calf, photographed in August 2013 in the waters of Caprera Canyon.

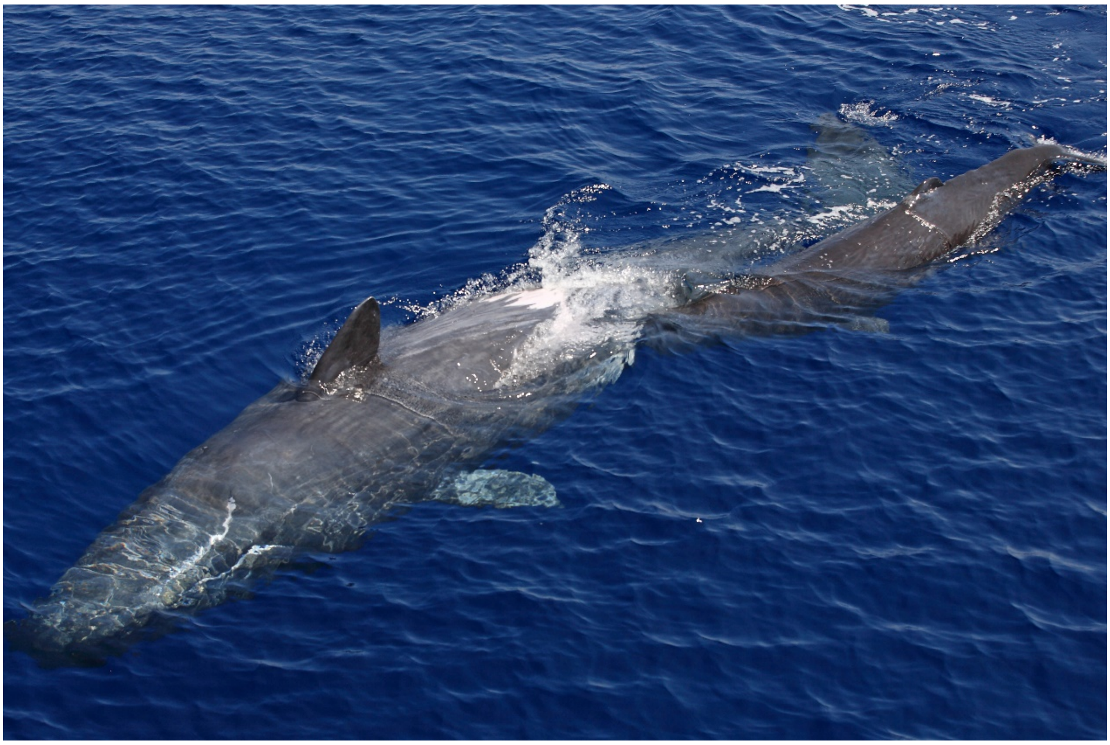

Common bottlenose dolphin *Tursiops truncatus*

Sightings summary:

Table S4.5 Sighting summary.

**No. Sightings** on/off effort	**No. individuals** on/off effort	**Group size** mean ± SD	**ER** mean ± SD	**Depth (m)** mean ± SD	**Slope (%)** mean ± SD
1/7	2/21	3 ± 1.8	0.1 ± 0.5	-102 ± 60	
2/12	18/67	5.6 ± 3.9	0.1 ± 0.4	154 ± 136	10 ± 5.7
0/19	0/100	5.3 ± 6.5		111 ± 98	
0/11	0/45	4.1 ± 3.2		73 ± 31	
0/8	0/26	3.3 ± 2.4		95 ± 50	
0/5	0/12	2.4 ± 1.5		91 ± 4	
0/11	0/35	3.2 ± 2		96 ± 48	
1/3	2/15	5 ± 4.4	0.2 ± 0.5	90 ± 9	
0/3	0/18	6 ± 2		97 ± 20	
4/79	22/339	4.3 ± 4.1	0 ± 0.3	105 ± 80	6.3 ± 5.8

ER: Encounter Rate as number of sightings per 100 km.

Sighting dynamics: Almost every bottlenose dolphin sighting made in this study occurred in their typical habitat, the neritic and coastal waters along the continental shelf. However, the species was rarely encountered in a different bathymetric domain: the continental slope.

Frequency of occurrence and relative abundance: The low encounter rate for this species can be explained by the fact that the coastal waters were ordinarily travelled in off–effort conditions. However, the occurrence of several off–effort sightings (n = 75, 95% of the total sightings of the bottlenose dolphin) over the whole study period, pointed out that the species is quite frequent and regularly inhabit the continental shelf waters off north–eastern Sardinia (mean depth = 105 m).

Group size: Bottlenose dolphins formed relatively small groups with a GSR of 2–12, and sightings were mainly recorded off–effort (Table 1).

Distribution and habitat preference: Bottlenose dolphin sightings were mainly recorded off–effort, and showed that this species is primarily restricted to the continental shelf, although it was also encountered in 2 deep–water sightings, on 27 July 2012 and 05 August 2013, respectively at a depth of 511 and 477 m, in the continental slope (Fig 5; Table 1). During the sightings of the bottlenose dolphins, several calves showed the foetal folds. The presence of calves and newborns (13% of the overall sightings), along with the documented observation of feeding behaviour both in association with the presence of fishing gears (e.g., gillnets) as well as hunting for prey in the absence of any fishing gear, pointed out the importance of the area for the bottlenose dolphin.

Behavioural observations: Travelling, feeding, resting, nursing, spy-hopping, socializing, tail slapping, milling, bow-riding and breaching were the observed behaviours of the bottlenose dolphins, recorded during the study period.

Threats and conservation: The Mediterranean subpopulation of bottlenose dolphin *T. truncatus* is listed as Least Concern in the IUCN Red List of threatened species [46]. The Bottlenose dolphin is the only cetacean species listed in Annex II of the EU Habitats Directive.

Other observations:

Relevant photographic documentation:

No relevant photographic documentation

Risso’s dolphin *Grampus griseus*

Sightings summary:

Table S4.6 Sighting summary.

**No. Sightings** on/off effort	**No. individuals** on/off effort	**Group size** mean ± SD	**ER** mean ± SD	**Depth (m)** mean ± SD	**Slope (%)** mean ± SD
2/2	16/16	8 ± 7.1	0.2 ± 0.8	-725 ± 40	5 ± 1.4
2/2	25/25	12.5 ± 0.7	0.2 ± 0.7	-819 ± 4	1.5 ± 0.7
0/0	0/0				
0/0	0/0				
2/2	16/16	8 ± 8.5	0.2 ± 0.6	-771 ± 24	5.5 ± 4.9
2/3	9/12	4 ± 1	0.3 ± 1	-835 ± 201	6.5 ± 7.8
1/1	10/10	10 ± 0	0.1 ± 0.6		
0/0	0/0				
0/0	0/0				
9/10	76/79	7.9 ± 4.9	0.1 ± 0.6	-796 ± 106	4.3 ± 3.9

ER: Encounter Rate as number of sightings per 100 km.

Sighting dynamics: Nevertheless, due to the small number of sightings (Table 1), the presence of calves in 40% of the sightings, and the observation of feeding behaviour, there may be a conservation concern for the Risso’s dolphin in the Caprera Canyon and adjacent waters. Furthermore, a photo-identification study investigated the movements of Risso’s dolphins across the northwestern Mediterranean Sea. Sightings recorded over several years by SEA ME Sardinia research group and Tethys Research Institute revealed one individual's long linear movement, totalling 341.2 km over 17 years between the Ligurian Sea and the Caprera Canyon area [47]. This suggests that the Caprera Canyon encompasses part of the Risso’s dolphin range in the central–western Mediterranean Sea.

Frequency of occurrence and relative abundance:

Group size: Risso’s dolphin was observed in pairs or up to 14 individuals. Calves were present in 40% of the overall sightings, and 69% of the calves showed foetal folds.

Distribution and habitat preference: In this study, the Risso’s dolphin was mainly encountered in the lower slope (depth range 696–1028 m) (Fig 5) and was observed forming groups with calves (30% of the overall sightings). Risso’s dolphins inhabit deep pelagic waters [48], typically at water depths ranging 200–2000 m [35], with a preference for steep shelf slopes and submarine canyons [30,49–51]. In the northern Pelagos Sanctuary (north–western Mediterranean Sea), a three-year study identified Risso’s dolphin hotspots primarily near the 1000 m isobaths [33]. The distribution of the Risso’s dolphin was modelled in the north–western Mediterranean Sea, suggesting that its main habitat was restricted to the shallowest, upper zone of the continental slope, at a mean depth of 638 m and a slope of 6.29 % [51].

Behavioural observations: Travelling, feeding, resting, nursing, spy-hopping, socializing, tail slapping, head-standing, bow-riding and breaching were the observed behaviours displayed from the Risso’s dolphins, recorded during the study period. During 2 sightings, 3 dolphins clearly displayed a feeding behaviour, inferred from the diving pattern of the different individuals, which involved repeated and alternating spin dives (according to [185]). They dived repeatedly in different points, surfaced facing in varying directions, and maintained an apparently uncoordinated behaviour (similar to what described in a behavioural study [52]), for around 30 minutes in each sighting.

Threats and conservation: The species was recently reassessed in the IUCN Red List of threatened species, and the Mediterranean subpopulation is currently listed as Endangered [53].

Other observations:

Relevant photographic documentation: Fig S4 6.A female-calf pair of Risso's dolphins observed in the study area.

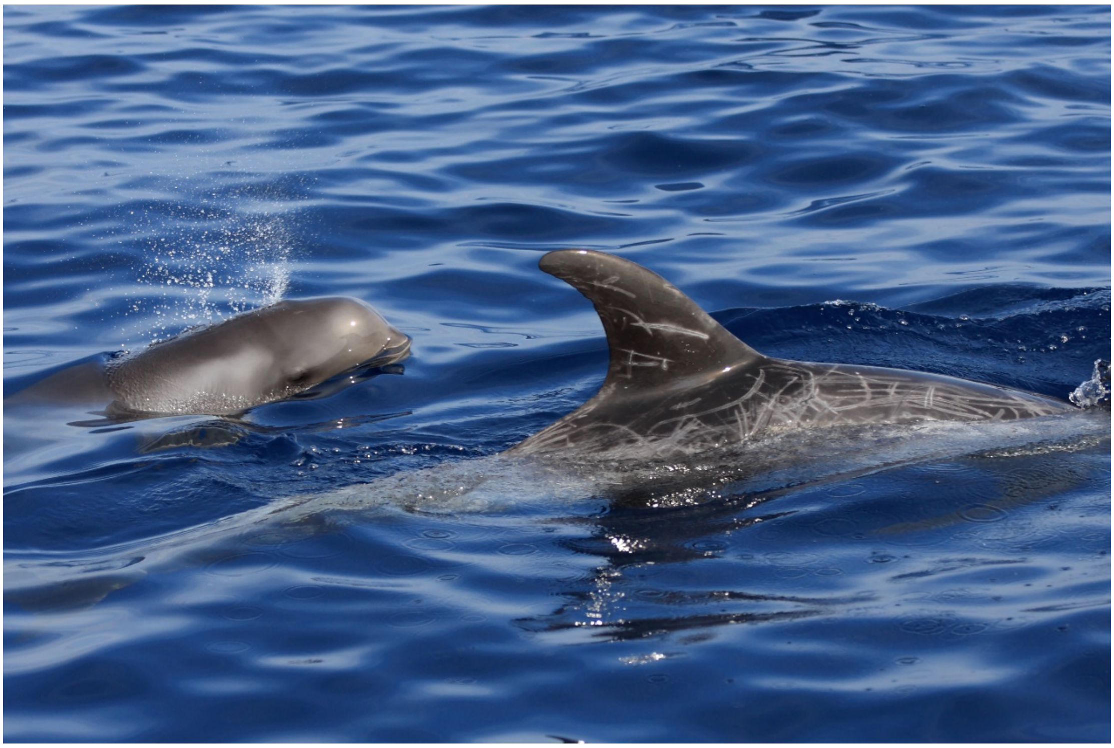

Common dolphin *Delphinus delphis*

Sightings summary:

Table S4.7 Sighting summary.

**No. Sightings** on/off effort	**No. individuals** on/off effort	**Group size** mean ± SD	**ER** mean ± SD	**Depth (m)** mean ± SD	**Slope (%)** mean ± SD
0/0	0/0				
0/0	0/0				
0/2	0/80	40 ± 28.3		-590 ± 37	1 ± 1.4
0/0	0/0				
0/1	0/35	35 ± 0			
0/1	0/25	25 ± 0			
0/3	0/54	18 ± 10.4		-618 ± 114	10.3 ± 9.7
0/1	0/60	60 ± 0			
0/0	0/0				
-/8	-/254	31.8 ± 19.2		-551 ± 145	8.1 ± 7.8

ER: Encounter Rate as number of sightings per 100 km.

Sighting dynamics: The common dolphin was observed only during off–effort mode, with its sightings partially overlapping those of the striped dolphin.

Frequency of occurrence and relative abundance:

Group size: The species was typically observed in medium to large groups (GSR = 12–60) composed by both juveniles and adults. Calves were observed in 3 out to the 8 sightings, with a total of 3 calves with evident foetal folds (photographic documentation in Fig S4 7). Although sightings were infrequent (n = 8), the large group size and the presence of calves with foetal folds in 38% of the sightings provide significant information about this species in the area.

Distribution and habitat preference: Information about the distribution and abundance of common dolphin in the Mediterranean Sea is still scarce. In the Ligurian and Tyrrhenian Sea, the preference of the common dolphin for slope waters was confirmed [35,54], obtaining a mean bottom depth of 674 m (SD = 463 m) in the western Mediterranean Sea [54]. In the Caprera Canyon area, the species was observed (off–effort) in 5 out of the 9–years involved in this study in the continental slope habitat, in relative shallow waters (mean depth = 551 m) and with a steep slope seabed. The distribution of common dolphin partially overlapped that of the striped dolphin, near the head of the Caprera Canyon system (Fig 5). Distribution, behavioural observations, and group composition data indicate this species may be using the slope habitat of the Caprera Canyon waters as a feeding and breeding area. Similarly, as was reported in this study, the common dolphin was rarely observed in most Mediterranean areas. For example, only 5 observations of this species were reported, yet with the largest groups observed in the study (mean GS = 76.8, GSR = 9–150) during a 5–years dedicated boat surveys (1986–1989) in the central Mediterranean Sea, none of which occurred in the Tyrrhenian Sea [9]. Over a 4–years study (1989–1992) conducted by ferry line–transect across the central Tyrrhenian Sea, 6 small groups (4–5 individuals) of common dolphin were recorded, with 2 observations of calves [8]. During fall 2013 (4 months, 3518 km of effort) in the eastern Sardinian margin and the eastern Pelagos Sanctuary [5] and over 1–year study in the central Tyrrhenian Sea [10], no sighting of common dolphin was reported. Based on small boat dedicated surveys carried out between 1988 and 2012 in the western Mediterranean, common dolphin was rarely observed off the French continental coast, but more frequent around Corsica, and quite frequent in waters off western Sardinia [54], obtaining an encounter rate of 1.4 groups/100 km in the northern Tyrrhenian Sea. Furthermore, during a 3–months survey in the waters off north–western Sardinia (western Mediterranean

Sea), only 2 sightings of this species were recorded [55]. A local population of common dolphin was studied in the waters off Ischia Island (Gulf of Naples, central–eastern Tyrrhenian Sea), obtaining a GS = 43.57 (n = 62). Results showed a significant steady decline, examining the decrease of the encounter rate over a period of 16 years (2000–2015) [56].
**Behavioural observations:** Nursing, socializing, tail slapping, bow-riding and breaching were the observed behaviours displayed from the species, recorded during the study period. On a few occasions, common dolphins were observed in association with striped dolphins (n = 3).
**Threats and conservation:** The common dolphin is listed as Endangered in the IUCN Red List, for the inner Mediterranean subpopulation [57].
**Other observations:**
**Relevant photographic documentation: Fig S4 7. A female-calf pair of common dolphins observed in the study area.** 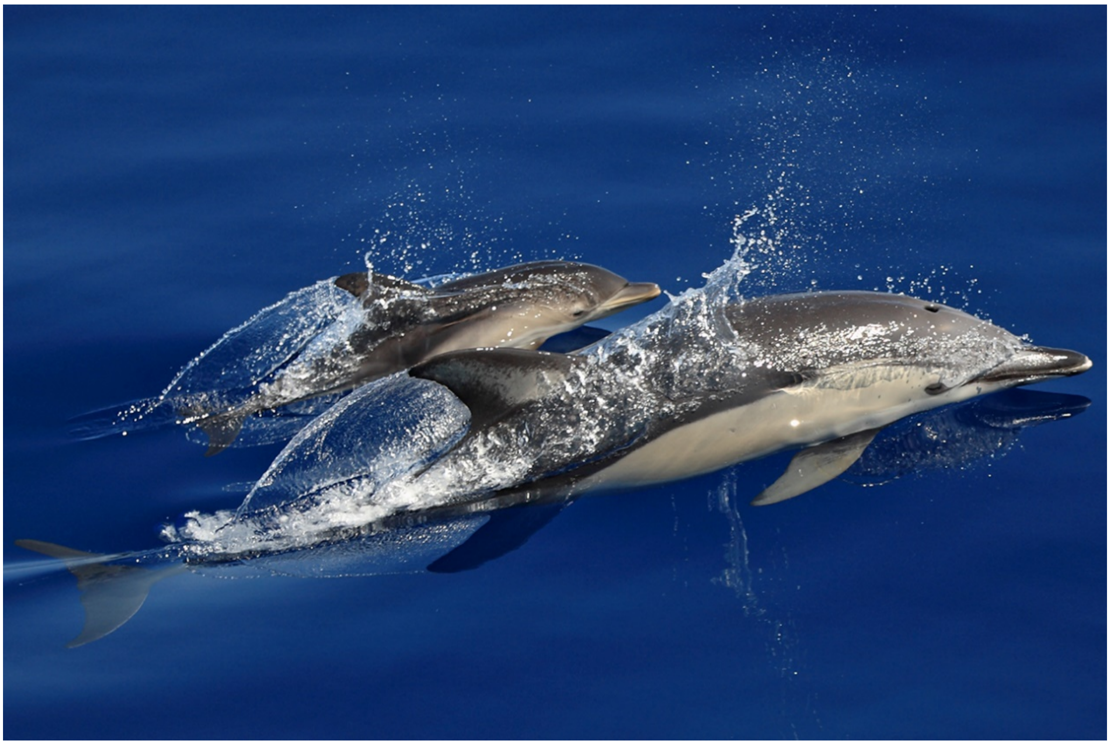

Sowerby's beaked whale *Mesoplodon bidens*
**Sighting summary:** Only a single sighting of Sowerby's beaked whale occurred in the Caprera Canyon during the surveyed period, on 17 June 2012 in a mixed species group with Cuvier's beaked whales. This, together with another sighting occurred 2 years earlier (2010), 44.4 km further north the Caprera Canyon area, represent, to date, the only free–ranging records of Sowerby's beaked whales in the Mediterranean Sea [58]. However, the species occurrence in the basin is still considered occasional and no further sightings were recorded until the end of the study period here presented.
**Sighting dynamics:**
**Frequency of occurrence and relative abundance:**
**Group size:** The individual was observed in a in a mixed species group with 3 Cuvier's beaked whales (*Ziphius cavirostris*).
**Distribution and habitat preference:**
**Behavioural observations:** For further information, see [58,59].
**Threats and conservation:**
**Other observations:**
**Relevant photographic documentation: Fig S4 8. The Sowerby's beaked whale sighted offshore NE Sardinia, in the Caprera Canyon waters, in June 2012.** 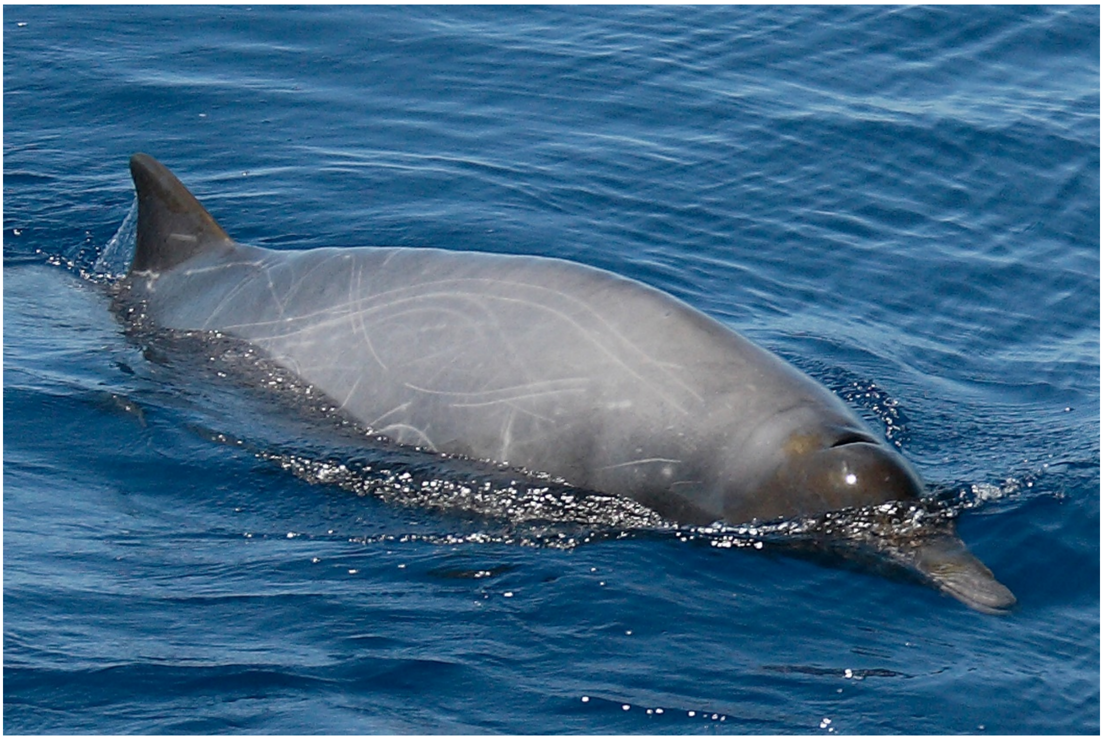

References

1. Gannier A. Summer distribution and relative abundance of delphinids in the Mediterranean Sea. Revue d'Ecologie, Terre et Vie. 2005;60: 223–238.
2. Mannocci L, Roberts JJ, Halpin PN, Authier M, Boisseau O, Bradai MN, et al. Assessing cetacean surveys throughout the Mediterranean Sea: A gap analysis in environmental space. Sci Rep. 2018;8: 3126.
3. ACCOBAMS. Estimates of Abundance and Distribution of Cetaceans, Marine Mega-Fauna and Marine Litter in the Mediterranean Sea from 2018–2019 Surveys. ACCOBAMS Survey Initiative Project Monaco; 2021.
4. Gannier AJ, Boyer G, Gannier AC. Recreational boating as a potential stressor of coastal striped dolphins in the northwestern Mediterranean Sea. Mar Pollut Bull. 2022;185: 114222. doi:https://doi.org/10.1016/j.marpolbul.2022.114222
5. Aïssi M, Arcangeli A, Crosti R, Daly Yahia M, Loussaief B, Moulins A, et al. Cetacean Occurrence and Spatial Distribution in the Central Mediterranean Sea Using Ferries as Platform of Observation. Russ J Mar Biol. 2015;41: 343–350. doi:10.1134/S1063074015050028
6. Arcangeli A, Marini L, Crosti R. Changes in cetacean presence, relative abundance and distribution over 20 years along a trans-regional fixed line transect in the Central Tyrrhenian Sea. Marine Ecology. 2013;34: 112–121. doi:10.1111/maec.12006
7. Forcada J, Aguilar A, Hammond PS, Pastor X, Aguilar R. Distribution and numbers of striped dolphins in the western Mediterranean Sea after the 1990 epizootic outbreak. Mar Mamm Sci. 1994;10: 137–150.
8. Marini L, Consiglio C, Angradi AM, Catalano B, Sanna A, Valentini T, et al. Distribution, abundance and seasonality of cetaceans sighted during scheduled ferry crossings in the central Tyrrhenian Sea: 1989–1992. Italian Journal of Zoology. 1996;63: 381–388.
9. Notarbartolo di Sciara G, Venturino MC, Zanardelli M, Bearzi G, Borsani F, Cavalloni B. Cetaceans in the central Mediterranean Sea: distribution and sighting frequencies. Italian Journal of Zoology. 1993;60: 131–138.

10. Arcangeli A, Campana I, Di Clemente J, Paraboschi M, Crosti R. Intra-Annual Changes in Cetacean Occurrence in the Western Mediterranean Sea. Annals of Marine Biology and Research. 2016;3: 1014.

11. Boisseau O, Lacey C, Lewis T, Moscrop A, Danbolt M, McLanaghan R. Encounter rates of cetaceans in the Mediterranean Sea and contiguous Atlantic. Journal of the Marine Biological Association of the United Kingdom. 2010;90: 1589–1599. doi:10.1017/S0025315410000342

12. Cañadas A, Sagarminaga R, García-Tiscar S. Cetacean distribution related to depth and slope in the Mediterranean waters off southern Spain. Deep-sea Research Part I-oceanographic Research Papers - DEEP-SEA RES PT I-OCEANOG RES. 2002;49: 2053–2073. doi:10.1016/S0967-0637(02)00123-1

13. Lauriano G. Stenella coeruleoalba (Mediterranean subpopulation). In: The IUCN Red List of Threatened Species 2022: e.T16674437A210833690. Accessed on 01 August 2023. [Internet]. 2022 [cited 21 Mar 2023]. Available: https://www.iucnredlist.org/species/16674437/210833690

14. Arcangeli A, Orasi A, Carcassi S, Crosti R. Exploring thermal and trophic preference of Balaenoptera physalus in the central Tyrrhenian Sea: A new summer feeding ground? Mar Biol. 2014;161: 427–436. doi:10.1007/s00227-013-2348-8

15. Cominelli S, Moulins A, Rosso M, Tepsich P. Fin whale seasonal trends in the Pelagos Sanctuary, Mediterranean Sea. J Wildl Manage. 2016;80: 490–499. doi:https://doi.org/10.1002/jwmg.1027

16. Panigada S, Notarbartolo di Sciara G, Panigada MZ, Airoldi S, Borsani JF, Jahoda M. Fin whales (Balaenoptera physalus) summering in the Ligurian Sea: distribution, encounter rate, mean group size and relation to physiographic variables. J Cetacean Res Manage. 2005;7: 137–145.

17. Tepsich P, Schettino I, Atzori F, Azzolin M, Campana I, Carosso L, et al. Trends in summer presence of fin whales in the Western Mediterranean Sea Region: new insights from a long-term monitoring program. Morán XAG, editor. PeerJ. 2020;8: e10544. doi:10.7717/peerj.10544

18. Edwards E, Hall C, Moore TJ, Sheredy C, Redfern J. Global distribution of fin whales Balaenoptera physalus in the post-whaling era (1980–2012). Mamm Rev. 2015;45: 197–214. doi:10.1111/mam.12048

19. Druon JN, Panigada S, David L, Gannier A, Mayol P, Arcangeli A, et al. Potential feeding habitat of fin whales in the western Mediterranean Sea: an environmental niche model. Mar Ecol Prog Ser. 2012;464: 289–306. Available: https://www.int-res.com/abstracts/meps/v464/p289-306/

20. Geijer CKA, Notarbartolo di Sciara G. Mysticete migration revisited: are Mediterranean fin whales an anomaly? Mammal Review. 2016;46: 284–296. doi:10.1111/mam.12069

21. Notarbartolo di Sciara G. Marine Mammals in the Mediterranean Sea: An Overview. Adv Mar Biol. 2016;75: 1–36.

22. Panigada S, Donovan GP, Druon J, Lauriano G, Pierantonio N, Pirotta E, et al. Satellite tagging of Mediterranean fin whales: working towards the identification of critical habitats and the focussing of mitigation measures. Sci Rep. 2017;7: 1–12. doi:10.1371/journal.pone.0022878

23. Panigada S, Gauffier P, Notarbartolo di Sciara G. Balaenoptera physalus (Mediterranean subpopulation). The IUCN Red List of Threatened Species 2021. Accessed on 01 August 2023: e.T16208224A50387979. 2021. Available: https://www.iucnredlist.org/species/16208224/50387979

24. Podestà M, Azzellino A, Cañadas A, Frantzis A, Moulins A, Rosso M, et al. Cuvier's beaked whale, Ziphius cavirostris, distribution and occurrence in the Mediterranean Sea: high-use areas and conservation threats. Advances in Marine Biology. Academic Press; 2016. pp. 103–140.

25. Cañadas A, Aguilar de Soto N, Aissi M, Arcangeli A, Azzolin M, Bearzi G, et al. challenge of habitat modelling for threatened low density species using heterogeneous data: the case of Cuvierâ s beaked whales in the Mediterranean. Ecol Indic. 2018.

26. Moulins A, Rosso M, Nani B, Würtz M. Aspects of the distribution of Cuvier's beaked whale (Ziphius cavirostris) in relation to topographic features in the Pelagos Sanctuary (north-western Mediterranean Sea). Journal of the Marine Biological Association of the United Kingdom. 2007;87: 177–186.

27. Arcangeli A, Campana I, Marini L, MacLeod C. Long-term presence and habitat use of Cuvier's beaked whale (Ziphius cavirostris) in the Central Tyrrhenian Sea. Marine Ecology. 2015;37: 269–282. doi:10.1111/maec.12272
28. Gannier A. Using existing data and focused surveys to highlight Cuvier's beaked whales favourable areas: A case study in the central Tyrrhenian Sea. Mar Pollut Bull. 2011;63: 10–17. doi:https://doi.org/10.1016/j.marpolbul.2010.03.037
29. D'Amico A, Bergamasco A, Zanasca P, Carniel S, Nacini E, Portunato N, et al. Qualitative correlation of marine mammals with physical and biological parameters in the Ligurian Sea. IEEE Journal of Oceanic Engineering. 2003;28: 29–43.
30. Azzellino A, Gaspari S, Airoldi S, Nani B. Habitat use and preferences of cetaceans along the continental slope and adjacent waters in the western Ligurian Sea. Deep Sea Research Part I: Oceanographic Research Papers. 2008;55: 296–323. doi:10.1016/j.dsr.2007.11.006
31. Cañadas A, Vázquez JA. Conserving Cuvier's beaked whales in the Alboran Sea (SW Mediterranean): Identification of high density areas to be avoided by intense man-made sound. Biol Conserv. 2014;178: 155–162.
32. Tepsich P, Rosso M, Halpin PN, Moulins A. Habitat preferences of two deep-diving cetacean species in the northern Ligurian Sea. Mar Ecol Prog Ser. 2014;508: 247–260.
33. Moulins A, Rosso M, Ballardini M, Würtz M. Partitioning of the Pelagos Sanctuary (north-western Mediterranean Sea) into hotspots and coldspots of cetacean distributions. Journal of the Marine Biological Association of the United Kingdom. 2008;88: 1273–1281.
34. MacLeod CD, Mitchell G. Key areas for beaked whales worldwide. Journal of Cetacean Research and Management. 2006;7: 309–322.
35. Gnone G, Bellingeri M, Airoldi S, David L, Di-Meglio N, Cañadas A, et al. Cetaceans in the Mediterranean Sea: Encounter Rate, Dominant Specie and Diversity Hotspots. Diversity (Basel). 2023;15: 321. doi:10.3390/d15030321
36. Arcangeli A, Campana I, Bologna M. Influence of seasonality on cetacean diversity, abundance, distribution and habitat use in the western Mediterranean Sea: Implications for conservation. Aquat Conserv. 2017;27. doi:10.1002/aqc.2758
37. CBD. Report of the Mediterranean Regional Workshop to Facilitate the Description of Ecologically or Biologically Significant Marine Areas. Montreal, QC, Canada: Convention

on Biological Diversity, UNEP/CBD/EBSA/WS/2014/3/4.2014. 2014. Available: https://www.cbd.int/doc/meetings/mar/ebsaws-2014-03/official/ebsaws-2014-03-04-en.pdf

38. Cañadas A, Notarbartolo di Sciara G. Ziphius cavirostris (Mediterranean subpopulation) (errata version published in 2021). In: The IUCN Red List of Threatened Species 2018: e.T16381144A199549199. Accessed on 21 March 2023 [Internet]. 2018 [cited 21 Mar 2023]. doi:https://dx.doi.org/10.2305/IUCN.UK.2018-2.RLTS.T16381144A199549199.en.

39. Mussi B, Miragliuolo A, Pace DS. Acoustic and behaviour of sperm whale nursery groups in the waters of Ischia, Italy. European Research on Cetaceans. 2005;19.

40. Pavan G. Continuous real-time monitoring with a deep underwater acoustic station. Noise spectra and biological sounds from the NEMO Test Site IWC SC/58 E. 2006;19: 1–4.

41. Frantzis A, Alexiadou P, Gkikopoulou KC. Sperm whale occurrence, site fidelity and population structure along the Hellenic Trench (Greece, Mediterranean Sea). Aquat Conserv. 2014;24: 83–102. doi:https://doi.org/10.1002/aqc.2435

42. Pace DS, Miragliuolo A, Mariani M, Vivaldi C, Mussi B. Sociality of sperm whale off Ischia Island (Tyrrhenian Sea, Italy). Aquat Conserv. 2014;24: 71–82.

43. Pirotta E, Carpinelli E, Frantzis A, Gauffier P, Lanfredi C, Pace DS, et al. Physeter macrocephalus (Mediterranean subpopulation). The IUCN Red List of Threatened Species 2021: e.T16370739A50285671. Accessed on 05 February 2023. 2021. Available: https://dx.doi.org/10.2305/IUCN.UK.2021-3.RLTS.T16370739A50285671.en.

44. Mazzariol S, Corazzola G, Graic JM, Centelleghe C, Marsili L, Terracciano G, et al. Mediterranean sperm whales unusual mortality event in 2019. Document SC68B/E/10/Rev1 presented at the 68th International Whaling Commission Scientific Committee meeting. 2020. Available: https://archive.iwc.int/pages/view.php?search=%21collection29937+&k=&modal=&display=list&order_by=title&offset=0&per_page=240&archive=&sort=DESC&restypes=&recentdaylimit=&foredit=&ref=17262

45. Fontanesi E, Leone M, Pintore L, Manconi R, Bittau L. First records of sperm whale (Physeter macrocephalus) with anomalously white pigmentation in the Tyrrhenian Sea. A living, Mediterranean Moby Dick? 32th European Cetacean Society Conference . 2018.

46. Natoli A, Genov T, Kerem D, Gonzalvo J, Holcer D, Labach H, et al. Tursiops truncatus (Mediterranean subpopulation). The IUCN Red List of Threatened Species in. 2021.

47. Remonato E, Airoldi S, Rosso M, Lanfredi C, Bittau L, Manconi R, et al. Where are the Risso’s Dolphins (Grampus griseus) of the North Western Mediterranean Sea? Study of Movements Within and Outside the Pelagos Sanctuary. 24th Annual Conference of the European Cetacean Society La Spezia, Italy, 6-10 April 2018. 2018.

48. Laran S, Pettex E, Authier M, Blanck A, David L, Dorémus G, et al. Seasonal distribution and abundance of cetaceans within French waters-Part I: The North-Western Mediterranean, including the Pelagos sanctuary. Deep Sea Research Part II: Topical Studies in Oceanography. 2017;141: 20–30.

49. Azzellino A, S P, Lanfredi C, M Z, Airoldi S, Notarbartolo di Sciara G. Predictive Habitat Models For Managing Marine Areas: Spatial And Temporal Distribution Of Marine Mammals Within The Pelagos Sanctuary (Northwestern Mediterranean Sea). Ocean Coast Manag. 2012;67: 63-74. doi:10.1016/j.ocecoaman.2012.05.024

50. Azzellino A, Airoldi S, Gaspari S, Lanfredi C, Moulins A, Podestà M, et al. Risso’s Dolphin, Grampus griseus, in the Western Ligurian Sea: Trends in Population Size and Habitat Use. Adv Mar Biol. 2016;75: 205–232. doi:10.1016/BS.AMB.2016.08.003

51. Praca E, Gannier A. Ecological niches of three teuthophageous odontocetes in the northwestern Mediterranean Sea. Ocean Science. 2008;4: 49–59.

52. Visser F, Keller OA, Oudejans MG, Nowacek DP, Kok ACM, Huisman J, et al. Risso’s dolphins perform spin dives to target deep-dwelling prey. R Soc Open Sci. 2021;8: 202320. doi:10.1098/rsos.202320

53. Lanfredi C, Arcangeli A, David L, Holcer D, Rosso M, Natoli A. Grampus griseus (Mediterranean subpopulation). In: The IUCN Red List of Threatened Species 2021: e.T16378423A190737150. Accessed on 04 August 2023 [Internet]. 2021 [cited 7 Aug 2023]. doi:https://dx.doi.org/10.2305/IUCN.UK.2021- 3.RLTS.T16378423A190737150.en

54. Gannier A. Present distribution of common dolphin Delphinus delphis in French Mediterranean and adjacent waters as obtained from small boat surveys. Aquat Conserv. 2021;31: 61–68. doi:https://doi.org/10.1002/aqc.2966

55. Lauriano G, Notarbartolo di Sciara G. The distribution of cetaceans off northwestern Sardinia. European Research on Cetaceans. 1995;9: 104–106.

56. Mussi B, Vivaldi C, Zucchini A, Miragliuolo A, Pace DS. The decline of short-beaked common dolphin (Delphinus delphis) in the waters off the island of Ischia (Gulf of Naples, Italy). Aquat Conserv. 2021;31: 87–100.

57. Bearzi G. Delphinus delphis (Mediterranean subpopulation). In: The IUCN Red List of Threatened Species 2012. Accessed on 21 March 2023: e.T134817215A19582908923 [Internet]. 2012 [cited 21 Mar 2023]. doi:10.2305/IUCN.UK.2012-1.RLTS.T134817215A195829089.en

58. Bittau L, Leone M, Gannier A, Gannier A, Manconi R. Two live sightings of Sowerby's beaked whale (Mesoplodon bidens) from the western Mediterranean (Tyrrhenian Sea). Journal of the Marine Biological Association of the United Kingdom. 2017/01/23. 2018;98: 1003–1009. doi:DOI: 10.1017/S0025315416001892

59. Bittau L, Gilioli V, Leone M, Costa G, Manconi R. First Mediterranean record of a free ranging Sowerby's beaked whale (Mesoplodon bidens) in a mixed species group with Cuvier's beaked whales (Ziphius cavirostris). 27th European Cetacean Society Conference, 8–10 April 2013, Setùbal (Portugal). 2013.
