# Supplementary figures and images for: The Caprera Canyon (north–eastern Sardinia): A hotspot of cetacean diversity in the western Mediterranean Sea
Source: PLoS One. 2025 Jul 9;20(7):e0326426. doi: 10.1371/journal.pone.0326426 (PMC12240396; doi:10.1371/journal.pone.0326426)

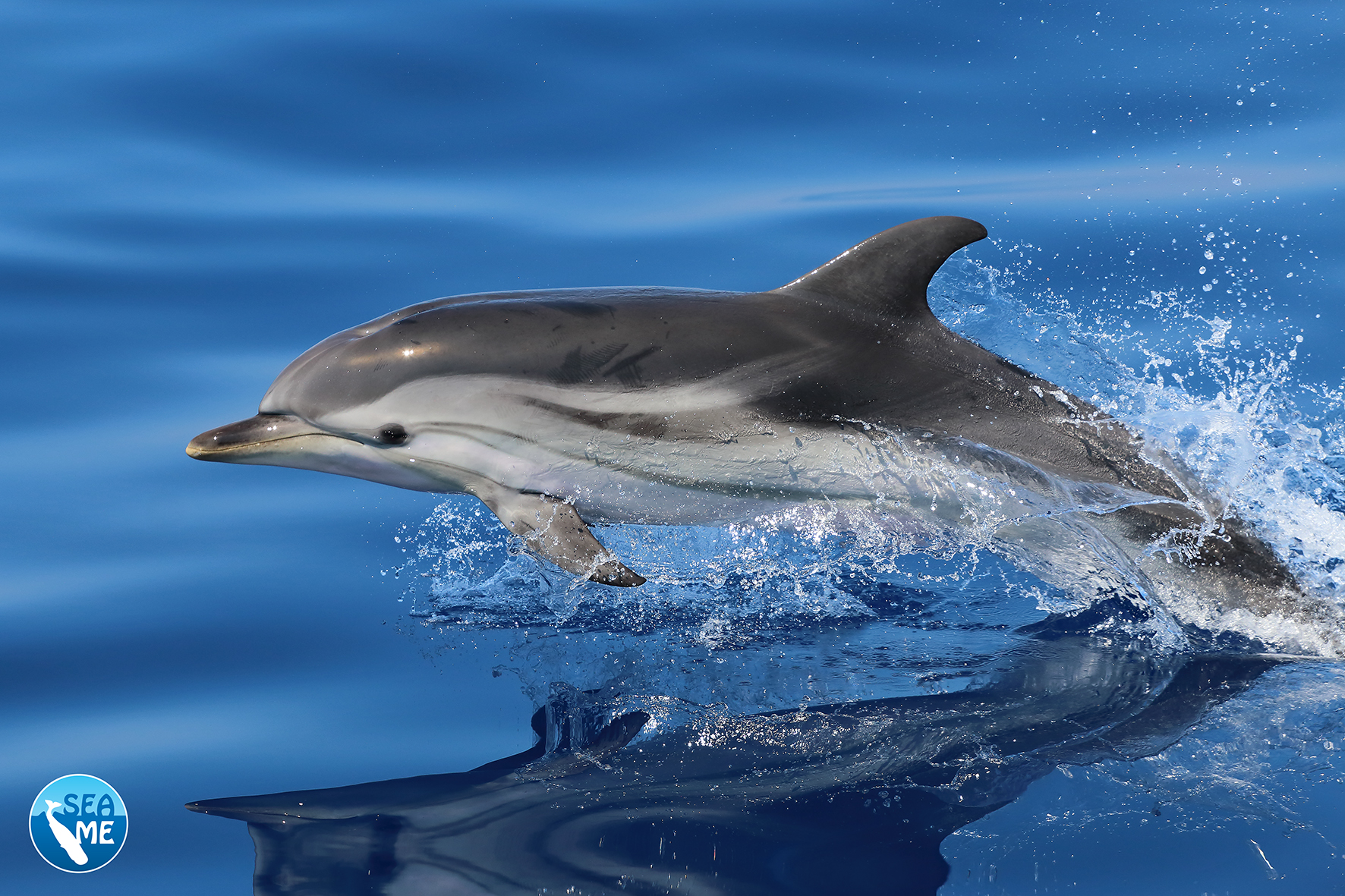

Supplement: S3 File — (JPG) [file pone.0326426.s005.jpg]
